# Unraveling the Enzymatic Basis of Wine “Flavorome”: A Phylo-Functional Study of Wine Related Yeast Species

**DOI:** 10.3389/fmicb.2016.00012

**Published:** 2016-01-20

**Authors:** Ignacio Belda, Javier Ruiz, Ana Alastruey-Izquierdo, Eva Navascués, Domingo Marquina, Antonio Santos

**Affiliations:** ^1^Department of Microbiology, Biology Faculty, Complutense University of MadridMadrid, Spain; ^2^Mycology Reference Laboratory, National Center for Microbiology, Instituto de Salud Carlos IIIMadrid, Spain; ^3^Agrovin, S.A.Ciudad Real, Spain

**Keywords:** microbial terroir, enological enzymes, non-*Saccharomyces*, phylo-functional study, targeted yeast selection

## Abstract

Non-*Saccharomyces* yeasts are a heterogeneous microbial group involved in the early stages of wine fermentation. The high enzymatic potential of these yeasts makes them a useful tool for increasing the final organoleptic characteristics of wines in spite of their low fermentative power. Their physiology and contribution to wine quality are still poorly understood, with most current knowledge being acquired empirically and in most cases based in single species and strains. This work analyzed the metabolic potential of 770 yeast isolates from different enological origins and representing 15 different species, by studying their production of enzymes of enological interest and linking phylogenetic and enzymatic data. The isolates were screened for glycosidase enzymes related to terpene aroma release, the β-lyase activity responsible for the release of volatile thiols, and sulfite reductase. Apart from these aroma-related activities, protease, polygalacturonase and cellulase activities were also studied in the entire yeast collection, being related to the improvement of different technological and sensorial features of wines. In this context, and in terms of abundance, two different groups were established, with α-L-arabinofuranosidase, polygalacturonase and cellulase being the less abundant activities. By contrast, β-glucosidase and protease activities were widespread in the yeast collection studied. A classical phylogenetic study involving the partial sequencing of 26S rDNA was conducted in conjunction with the enzymatic profiles of the 770 yeast isolates for further typing, complementing the phylogenetic relationships established by using 26S rDNA. This has rendered it possible to foresee the contribution different yeast species make to wine quality and their potential applicability as pure inocula, establishing species-specific behavior. These consistent results allowed us to design future targeted studies on the impact different non-*Saccharomyces* yeast species have on wine quality, understanding intra and interspecific enzymatic odds and, therefore, aiming to predict the most suitable application for the current non-*Saccharomyces* strains, as well as the potential future applications of new strains. This work therefore contributes to a better understanding of the concept of wine microbiome and its potential consequences for wine quality, as well as to the knowledge of non-*Saccharomyces* yeasts for their use in the wine industry.

## Introduction

Microorganisms coexist and interact in many environments and processes, and this fact is of practical relevance for both the environmental and industrial fields (Ivey et al., 2013). Grape musts naturally contain a mixture of yeast species, and wine fermentation is not a “single-species” process (Fleet, 1990). Despite the dominance of *Saccharomyces cerevisiae* in fermentation, which is expected and welcomed to avoid stuck and sluggish fermentations, the indigenous non-*Saccharomyces* yeasts, already present in the musts, play a critical role during the early stages of fermentation. While these yeast species are not the ones mainly responsible for alcoholic fermentation, they can release a wide variety of hydrolytic enzymes depending on their diversity (Jolly et al., 2014). Non-*Saccharomyces* yeasts were originally held responsible for microbe-related problems in wine production due to their isolation from spoiled wines. However, in recent years both empiric and scientific knowledge has emerged concluding that, in some cases, higher microbial diversity improves wine complexity.

The concept of vineyard and wine microbiome has been addressed in recent years, obtaining extensive and meaningful results on the microbial complexity of the fermentation process (Liu et al., 2015). These population studies, carried out by both classical molecular methods and metagenomics, are currently ongoing to better understand and establish the concept of “microbial terroir” (Bokulich et al., 2013, 2014; Gilbert et al., 2014). Considering that a wide variety of yeast species have been identified in different scientific studies (Bisson and Joseph, 2009; Barata et al., 2012), the role of all these yeast species and their intraspecific variations need to be known. There is an intense debate over the pertinence of the concept of microbial terroir in vineyards and wine fermentation. Several factors have been described as determinants of microbial diversity in enological environments. Robust results reported by Bokulich et al. (2014) and Wang et al. (2015) have concluded that grape-associated microbial biogeography is non-randomly associated with regional, varietal and climatic factors across multi-scale viticultural areas. However, this concept should be studied in depth, encompassing a strain-typing level and its final influence on wine quality.

A non-*Saccharomyces* strain was first used intentionally in wine fermentation in the 1960s, when Cantarelli (1955) significantly reduced the volatile acidity of wines by using selected *Torulaspora delbrueckii* strains. Nowadays, there is a wide variety of current and expected applications of non-*Saccharomyces* yeasts whose metabolic heterogeneity not only allows overcoming certain shortcomings detected in most *S. cerevisiae*, but also enables the development of innovative fermentation processes to obtain wines with new properties in sensorial, technological and safety aspects.

Apart from reducing volatile acidity in wines (Moreno et al., 1991; Renault et al., 2009), other specific applications have been attributed to certain wine yeast species, such as alcohol reduction (Contreras et al., 2014), modulation of acidity (Gobbi et al., 2013; Benito et al., 2015), increased glycerol content (Ciani and Ferraro, 1998; Soden et al., 2000), mannoprotein release (Belda et al., 2015), and the modulation of wine aroma profiles and other microbial products (reviewed by Jolly et al., 2014). In addition to fermentative aromas, mainly dependent on *S. cerevisiae* metabolism, non-*Saccharomyces* yeasts have long been described as a useful tool for revealing the varietal profile of certain grape varieties, whose aroma-determinant components are usually found as odorless conjugated precursors (Gunata et al., 1990; Tominaga et al., 1998). Trace amounts of terpenes and thiols could be present in grapes in a free form, although during fermentation yeasts may also release them from their corresponding odorless precursors. The cleavage of terpenic glycosides is dependent on the hydrolytic activity of glycosidases (Mateo and Di Stefano, 1997) and β-lyases for cysteine-conjugated thiols (Swiegers et al., 2009).

However, the improvement of the aromatic properties of wine is not the only aspect dependent on the enzymatic properties of yeasts, as other sensorial and technological features can be enhanced by other hydrolytic activities. Pectinolytic enzymes (mainly polygalacturonase) are widely used in enology to help degrade the plant cell wall polysaccharides of the grape skin and pulp. They can also help to improve clarification and filterability processes, releasing more color and flavor compounds entrapped in the grape skin, and facilitating the release of phenolic compounds (Lang and Dornenburg, 2000; Van Rensburg and Pretorius, 2000). Finally, the use of proteases in winemaking is not a widely extended practice at the present time, with bentonite being used more frequently to solve protein haze problems. The use of bentonite usually impairs the sensorial properties of wines, so the use of proteases for this purpose may be a potential solution (Marangon et al., 2012).

On the other hand, the presence of sulfite reductase in wine yeast strains is responsible for the production of hydrogen sulfide in wine fermentations, with the consequent appearance of the characteristic rotten egg off-flavor (Swiegers and Pretorius, 2007).

This paper explores the knowledge established between the concepts of wine microbiome and microbial terroir, linking the phylogenetic data provided with the enzymatic characteristics determined in a wide yeast collection. These results have allowed us to establish a general enzymatic phenotypical characterization of several wine-related yeast species and their intraspecific variability, predicting the impact of yeast microbiome on wine flavor. Thus, since the wine microbial terroir has been defined as the distinctive autochthonous microbiome of a wine region and it has been experimentally demonstrated as a determining feature of wine qualities (Bokulich et al., 2014), this work provides a compelling basis to understand the influence of these microbial differences on the wine flavor identity, developing the new concept of wine yeast flavorome and also providing some of its enzymatic basis.

## Materials and methods

### Grape samples and yeast isolation

Grape samples were collected from three different Spanish wine appellations: Tierra de León (vineyard named in this study as G), Ribera del Duero (vineyards named as PDC and EM) and Rueda (vineyard named as O). G is a young (20–40 years old) vineyard with vines of the Prieto Picudo variety; the PDC and EM vineyards are between 25 and 91 years old, with vines of the Tempranillo variety; and O is an ancient vineyard with pre-Phylloxera vines between 100 and 200 years old of the Verdejo variety, and also involves biodynamic agricultural practices. Representative samples were taken by analyzing a variety of different sample points depending on the particular agronomical heterogeneity of each vineyard. Three samples points were selected in vineyard G, 10 in vineyard PDC, 5 in vineyard EM and 9 in vineyard O.

Seventy-three yeasts were isolated from vineyard G during the 2012 harvest; 450 yeasts were isolated from vineyards PDC and EM during the 2013 and 2014 harvests; and finally, 247 yeasts were isolated from vineyard O during the 2013 and 2014 harvests (Table S1).

For the isolation of non-*Saccharomyces* yeasts, grape samples weighing about 0.5 kg were taken from healthy grape bunches. After pressing, to reduce the number of ubiquitous *A. pullulans* and basidiomycetous species of no interest to the enological objectives of this work, grape musts were incubated overnight at 20°C. A suitable diluted aliquot of grape must was then spread onto a lysine agar medium (Oxoid) plates at 28°C for 48 h. As stated above, 770 discrete colonies were isolated, and then restreaked on the same medium to obtain pure cultures that were cryopreserved and included in a yeast collection.

These yeast isolates were identified by partial sequencing of the 26S large subunit rRNA gene. Total genomic DNA was extracted using the isopropanol method (Querol et al., 1992), and the DNA for sequencing was amplified by using an Eppendorf Mastercycler, with forward NL-1 primer (5′-GCA TAT CAA TAA GCG GAG GAA AAG-3′) and reverse NL-4 primer (5′-GGT CCG TGT TTC AAG ACG G-3′) (Kurtzman and Robnett, 1997). The sequences obtained to identify yeasts were analyzed and compared by BLAST-search (http://blast.ncbi.nlm.nih.gov/Blast.cgi). Finally, sequences were deposited in the GenBank database (http://www.ncbi.nlm.nih.gov/genbank/) with the accession numbers listed in Table S1.

### Phylogenetic tree analysis

Phylogenetic analyses were conducted with InfoQuest FP Software (version 4.5 Bio-Rad Laboratories, Madrid, Spain). The clustering was performed following the Neighbor joining (NJ) method, with Kimura two-parameter correction.

### Culture media and enzymatic screening procedures

#### Glycosidase activities

β-Glucosidase activity was evaluated as reported by Villena et al. (2005) on a medium containing 0.5% cellobiose (4- O-β-D-glucopyranosyl-D-glucose), 0.67% yeast nitrogen base (Difco) and 2% agar. This medium was adjusted to pH 3.5 as follows. The components of the medium were sterilized separately to avoid agar hydrolysis. Agar and cellobiose were autoclaved, and the yeast nitrogen base was adjusted to pH 3.5 with HCl and then filtered (0.22 μm). Both fractions were subsequently mixed when the agar solution was around 60°C. A loop full of each yeast strain was spread onto the medium surface and incubated at 28°C for 3 days. Any significant growth of the colonies indicated the presence of β-glucosidase activity. A positive control (*Rhodotorula glutinis* CECT 10143) and a negative one (*Torulaspora delbrueckii* CECT 10676) were used as reference for growth determinations.

Additionally, β-D-xylosidase and α-L-arabinofuranosidase activities were evaluated using the corresponding methylumbelliferyl-conjugated substrates (methylumbelliferyl-β-D-xylopyranoside (MUX) and methylumbelliferyl-α-L-arabinofuranosidase (MUA), respectively; Sigma-Aldrich), according to the method described by Manzanares et al. (1999), with slight modifications for their development in 96-well microplates. Methylumbelliferone release was measured by detecting fluorescence using a Varioskan Flash Mutimode Reader (Thermo Scientific) with an excitation wavelength at 355 nm and emission at 460 nm. Once again, *R. glutinis* CECT 10143 and *T. delbrueckii* CECT 10676 were used as positive and negative controls, respectively.

#### β-lyase activity

β-Lyase activity was evaluated on a medium containing the following: 0.1% S-methyl-L-cysteine (Sigma-Aldrich), 0.01% pyridoxal-5′-phosphate (Sigma-Aldrich), 1.2% Yeast Carbon Base (Difco, Detroit, MI, USA) and 2% agar. This medium was adjusted to pH 3.5 and sterilized as described above to avoid agar hydrolysis. The agar solution was autoclaved, and all the other components were adjusted to pH 3.5 with HCl and filtered (0.22 μm), then both fractions were mixed when the agar solution was around 60°C. Any significant growth of the colonies after 48–72 h indicated the presence of β-lyase activity (Patent pending). *T. delbrueckii* CECT 10676 and *R. glutinis* CECT 10143 were used as positive and negative controls, respectively.

#### Pectinase activities

Yeast isolates were screened for polygalacturonase activity in a polygalacturonate agar medium containing 1.25% polygalacturonic acid (Sigma), 0.67% yeast nitrogen base (YNB, Difco), 1% glucose and 2% agar, adjusted to a final pH 3.5, as previously described (Strauss et al., 2001), with slight modifications. Agar was sterilized separately by autoclaving, and all the other components were adjusted to pH 3.5 and boiled. Both solutions were mixed when agar reached a temperature of around 60°C. *Metschnikowia pulcherrima* CECT 11202 and *Lachancea thermotolerans* CECT 1951 were used as positive and negative controls, respectively.

#### Protease activities

Protease activity was evaluated on YPD plates (containing 1% yeast extract, 2% peptone, 2% glucose, and 2% agar) with 2% skim milk powder (Sigma-Aldrich). The plates were incubated for 5 days at 30°C. A clear zone around the colony identified protease activity (Strauss et al., 2001). *Wickerhamomyces anomalus* PYCC 2495 and *T. delbrueckii* CECT 10676 were used as positive and negative controls, respectively.

#### Cellulase activities

Cellulase production was determined on YPGE plates (containing 1% yeast extract, 2% peptone, 3% glycerol, and 2% ethanol) with 0.4% carboxymethylcellulose, as previously described (Teather and Wood, 1982*). Aureobasidium pullulans* CECT 2660 and *T. delbrueckii* CECT 10676 were used as positive and negative controls, respectively.

#### Sulfite reductase activity

Hydrogen sulfide production was evaluated by using a modification of the lead acetate method (Linderholm et al., 2008) described by Belda et al. (2015) for its use in 96-well microplates. Briefly, this method detects volatile H_2_S in the headspace of a culture medium containing 1.17% yeast carbon base (Difco), 4% glucose anhydrous, and 0.5% ammonium sulfate. Yeasts were grown at 28°C for 3 days in 96-well microplates containing 200 μl of medium with orbital agitation (200 rpm). Hydrogen sulfide formation was initially detected by using paper strips (Whatman filter paper) that had been previously embedded with a 0.1 M lead acetate solution and allowed to dry at 65°C for 10 min and deposited over microplate wells. Hydrogen sulfide formation was qualitatively measured based on the degree of blackening of the lead acetate strip, and quantitatively estimated by densitometric measurement of the color intensity (Software “My Image Analysis v1.1” Thermo Scientific). *R. glutinis* CECT 10143 and *T. delbrueckii* CECT 10676 were used as positive and negative controls, respectively.

#### Statistical analysis of enzymatic data

Enzymatic activity was coded on a scale from 1 (no activity) to 5 (highest activity) and loaded into InfoQuest FP Software (version 4.5 Bio-Rad Laboratories, Madrid, Spain) as a character type. A similarity matrix was calculated using the Unweighted Pair Group Method with Arithmetic Mean (UPGMA). Groups were assigned according to the identification of the strains by 26S analysis. Group separation was calculated with the Jackknife method. Principal Components Analysis (PCA) was performed with InfoQuest FP Software.

The species distribution per sample site was introduced into R program (R Core Team, 2013). The function vegdist from the package vegan version 2.2-1 (Oksanen et al., 2015) was used to calculate a dissimilarity matrix between samples.

## Results

### Description of yeast populations

**Figure 1:**
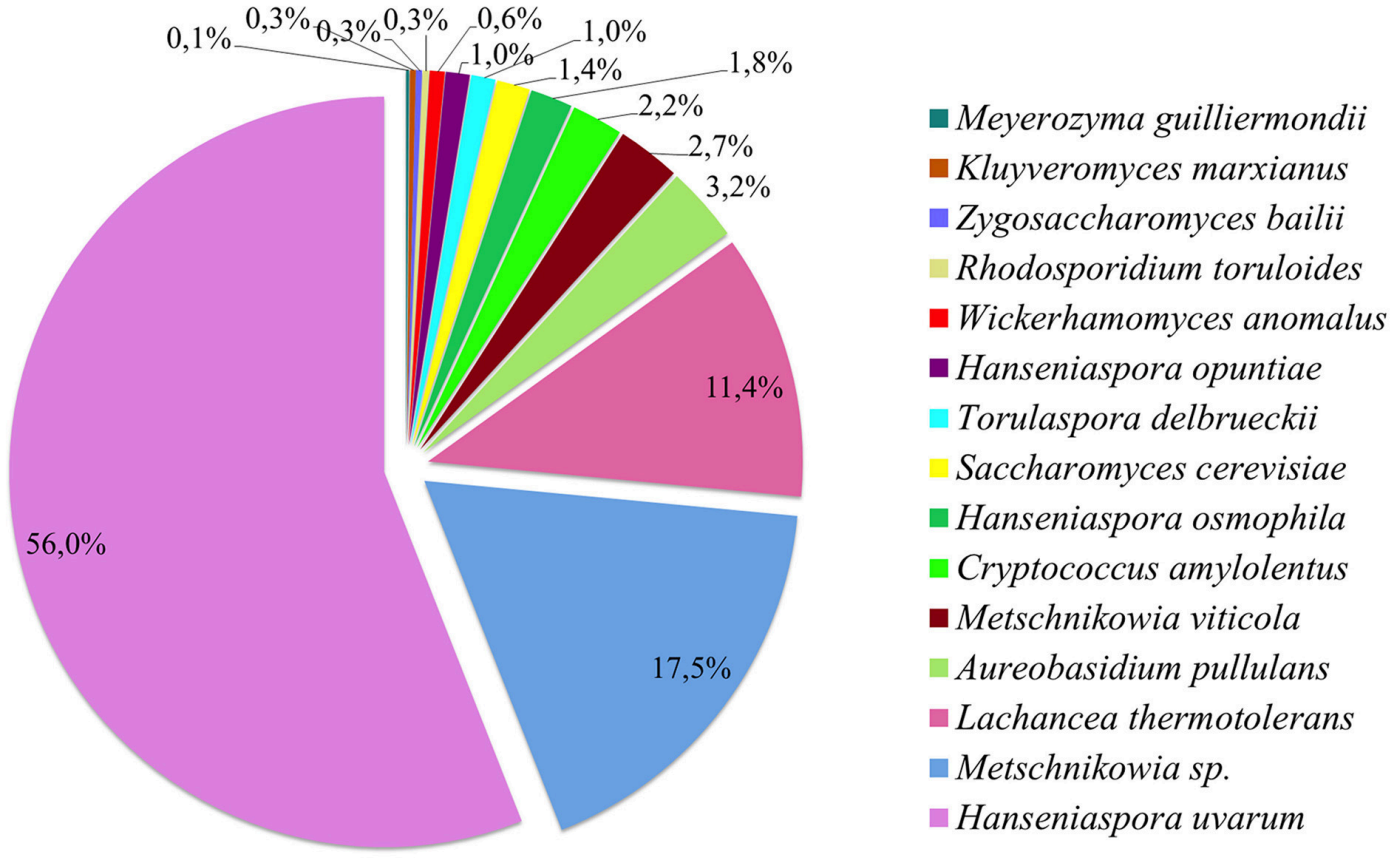
**Population distribution across the 770 yeast isolates**.

In this work, 770 yeast isolates from grape musts of different origins were identified by partial sequencing of the 26S rRNA gene (Table S1). Fifteen different species were found among the yeast collection studied here (Figure 1), which consisted of a wide range of yeast species usually found in vineyards, and mostly having been reported to be of enological interest (Fleet, 2008; Jolly et al., 2014). *Hanseniaspora uvarum* was the most abundant species, making up more than half of the total isolates, followed by *Metschnikowia* sp. (comprising *M. pulcherrima* and *M. fructicola*) and *Lachancea thermotolerans*, with the other 12 yeast species only present at levels of less than 4% (Figure 1). In spite of this small diversity of species, the high sample size (770 isolates) allowed us to conduct a functional analysis of the yeast collection in question. Considering the complete yeast collection used here, a phylogenetic analysis of the 770 isolates, belonging to 15 yeast species identified on the basis of rDNA 26S sequences, was carried out in order to confirm the success of the molecular identification process (Figure S1). It should be noted that *M. fructicola* and *M. pulcherrima* could not be properly differentiated by 26S sequence analysis (Guzmán et al., 2013), and are henceforth referred to here as *Metschnikowia* sp.

**Figure 2:**
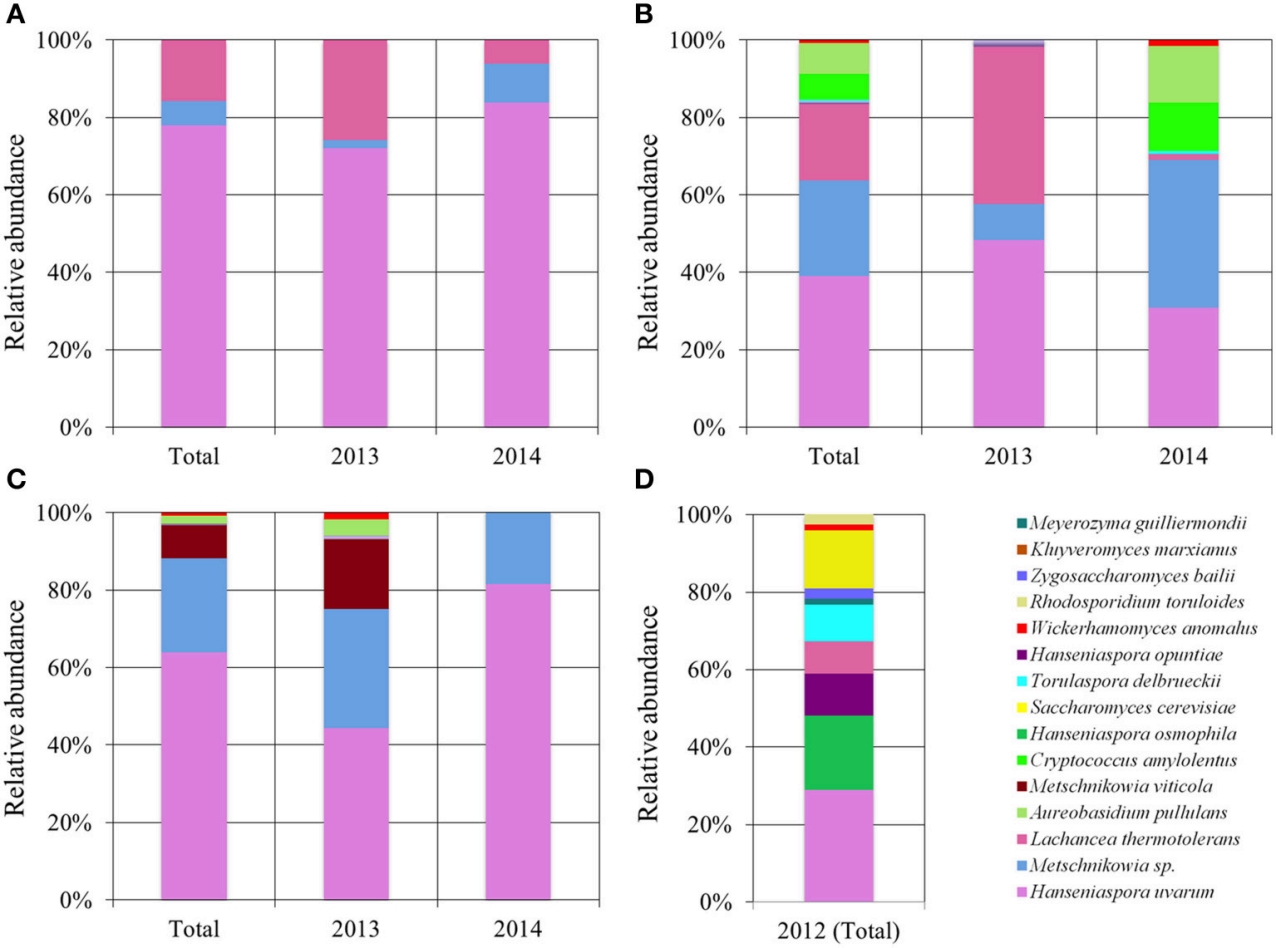
**Total and vintage-specific population distribution from the four sampled vineyards**. **(A)** Population distribution of EM vineyard. **(B)** Population distribution of PDC vineyard. **(C)** Population distribution of O vineyard. **(D)** Population distribution of G vineyard.

Notable differences between the diversity and richness of yeast species in the different vineyards sampled were observed (Figure 2, Table S3). Furthermore, some differences could be perceived between yeast populations of different vintages from the same vineyard. Particular note should be taken of the low diversity of yeast species in the EM vineyard, which had only three yeast species, all of which were identified in both the 2013 and 2014 vintages, with *H. uvarum* accounting for more than three quarters of the total of 196 isolates, followed by *L. thermotolerans* and *Metschnikowia* sp. (Figure 2A).

In the case of the PDC vineyard (Figure 2B), a total of 254 yeast isolates, comprising eight species, were obtained. *H. uvarum, Metschnikowia* sp. and *L. thermotolerans* were once again the most dominant species (39, 24.8, and 19.7% of the total population, respectively). However, in this case, significant differences could be observed between vintages. There was a significant decrease in *L. thermotolerans* isolates in the 2014 vintage, and there was a higher diversity. The other species identified were *Aureobasidium pullulans, Cryptococcus amylolentus, Wickerhamomyces anomalus, Kluyveromyces marxianus*, and *Torulaspora delbrueckii*, jointly accounting for less than 16.6% of the PDC population and 5.4% of the total population.

Similar diversity was observed in the O vineyard, with six yeast species being identified among the 247 isolates (Figure 2C). *H. uvarum* was again the most abundant, accounting for 64.4% of the total, with the key observation being the low abundance of *L. thermotolerans* (one of 247 isolates). It should be noted that in this vineyard *M. viticola* was identified as an additional *Metschnikowia* species. Contrary to what was observed in the PDC vineyard, a higher diversity was found in the 2013 vintage, when compared to 2014, when only *H. uvarum* and *Metschnikowia* sp. were isolated.

The G vineyard comprised 10 yeast species (nine non-*Saccharomyces* species along with some *Saccharomyces cerevisiae* isolates). *Hanseniaspora* genus was distributed among isolates of three species: *H. uvarum* (28.8%), *H. osmophila* (19.2%), and *H. opuntiae* (11%) (Figure 2D). Apart from *Hanseniaspora* species and *L. thermotolerans*, in the other vineyards the other five non-*Saccharomyces* species were either not isolated (*Meyerozyma guilliermondii, Zygosaccharomyces bailii*, and *Rhodosporidium toruloides*) or rarely isolated (*W. anomalus* and *T. delbrueckii*). In this case, the absence of isolates from different vintages made it impossible to establish any population trends. Finally, contrary to what was expected due to the use of a lysine medium, 11 yeast isolates were identified as *S. cerevisiae*; nevertheless, they were not removed from the collection, but instead used as a comparative control for the enzymatic study.

### Phylo-functional study

To address a targeted use of non-*Saccharomyces* species in the wine industry, it is required a better understanding of their specific metabolic properties and their strain-dependent features. Different yeast species have been reported to modulate wine flavor and aroma, in part because of their enzymatic properties (Hernández-Orte et al., 2008; Maturano et al., 2015). The main aim of this work was to robustly establish the wine-related enzymatic profile of a large collection of wine yeasts.

**Figure 3:**
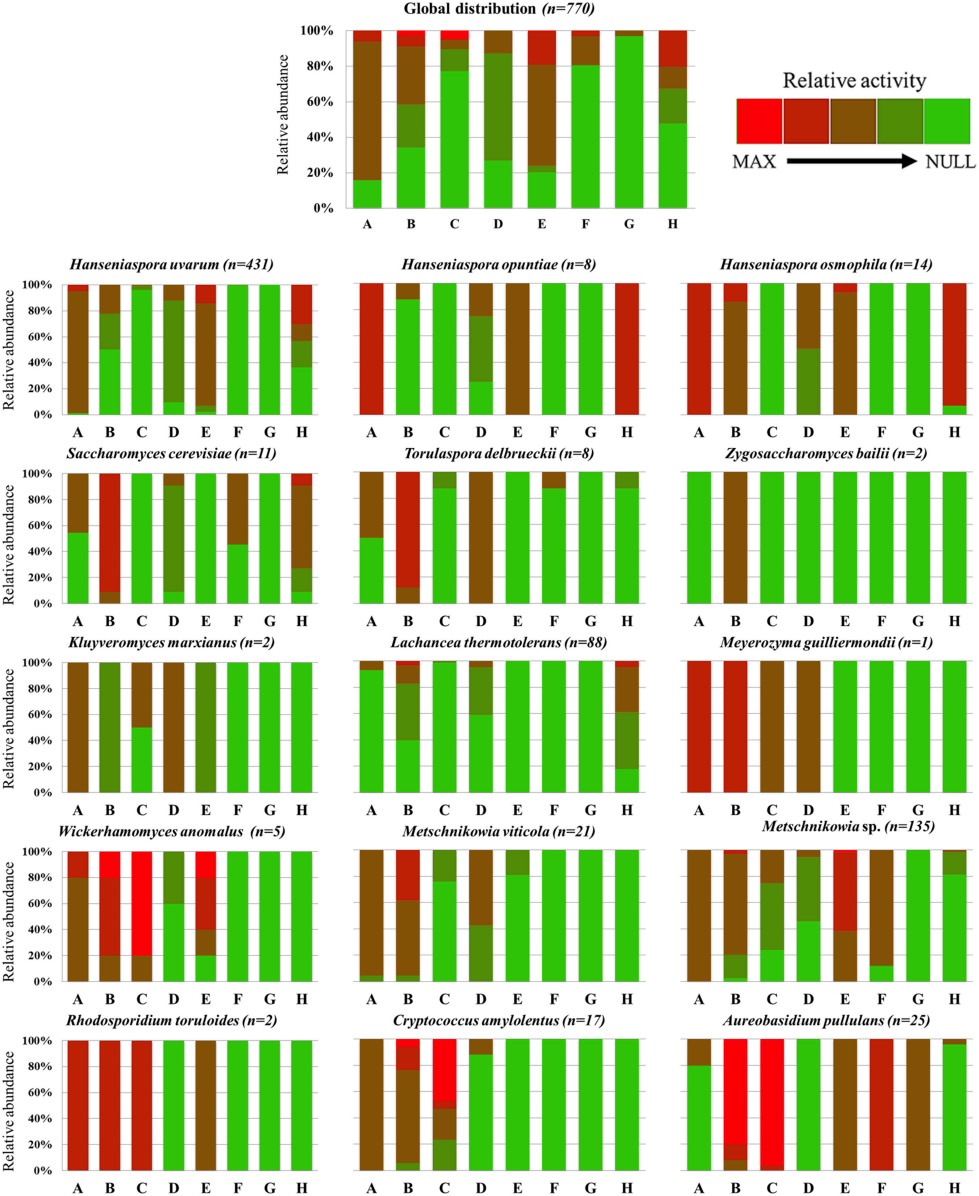
**Abundance and distribution of enzymatic activities among the total yeast collection, individually considering the 15 yeast species identified**. The eight enzymatic activities evaluated were: (A) β-glucosidase; (B) β-D-xylosidase; (C) α-L-arabinofuranosidase; (D) β-lyase; (E) Protease; (F) Polygalacturonase; (G) Cellulase; (H) Hydrogen sulfide production. Enzymatic activity was determined on a scale from 1 (no activity) to 5 (highest activity) corresponding to a progressive color code from green to red.

A combined analysis of phylogenetic and enzymatic data (β-glucosidase, α-L-arabinofuranosidase, β-D-xylosidase, β-lyase, protease, polygalacturonase (pectinase), cellulase, and sulfite reductase) was performed to observe whether there were any overall differences in enzyme abundances and their presence among different phylogenetic groups, inferring species-specific behaviors (Figure 3, Figure S1). In this context, two different groups of highly and less abundant enzymes could be established, highlighting α-L-arabinofuranosidase, polygalacturonase and cellulase as the least abundant activities and, on the other hand, β-glucosidase and protease as the most widespread activities throughout the yeast collection studied.

Figure 3 shows the overall abundance and activity level of the different enzymes studied in the 770 yeast isolates, and their distribution among the 15 species identified. β-Glucosidase was widespread among wine yeast species. All the strains of *Z. bailii* and *L. thermotolerans* were observed to be β-glucosidase negative, whereas most of the strains belonging to *A. pullulans, T. delbrueckii* and *S. cerevisiae* were also found to be β-glucosidase negative, without any species-specific behavior. On the other hand, note should be taken of the activity of *H. osmophila, H. opuntiae, M. guilliermondii*, and *R. toruloides* (Figure 3, Figure S1). Regarding the other two glycosidases, the abundance of β-D-xylosidase and α-L-arabinofuranosidase was found to be of medium and low, respectively. Special mention should be made of the production of β-D-xylosidase in *S. cerevisiae, T. delbrueckii, M. guilliermondii, W. anomalus, R. toruloides*, and *A. pullulans*, with the production of α-L-arabinofuranosidase being only noteworthy in the three latter species, as well as in *C. amylolentus.* Overall, a glycosidase-active cluster could be observed in the basidiomycetous group (*C. amylolentus* and *R. toruloides*), together with the yeast-like fungus *A. pullulans*, all of them located at the bottom of the phylogenetic tree (Figure S1).

β-Lyase activity was widespread, albeit in most cases with moderate activity throughout the isolates. Only *T. delbrueckii, M. guilliermondii*, and *K. marxianus* had a wholly positive specific behavior.

Protease activity was, together with β-glucosidase, the most abundant activity in the yeast population studied. However, 40% of the yeast species (six out of 15) had no protease activity. This apparent contradiction can be explained by the small representation these species have in the total number of yeast isolates. It should be mentioned that protease activity was fully absent in the phylogenetically related species *S. cerevisiae, Z. bailii*, and *T. delbrueckii*, as well as in *L. thermotolerans, M. guilliermondii*, and *C. amylolentus* (Figure 3).

On the other hand, pectinase and cellulase activities had a restricted distribution, with pectinase having only a significant presence in *Metschnikowia* sp. and *A. pullulans*, and cellulase only in *A. pullulans*. Apart from that, almost half of *S. cerevisiae* and a few *T. delbrueckii* isolates had pectinase activity. It should be mentioned that protease and pectinase activities are the main phenotypic differences between *M. viticola* and the other two *Metschnikowia* species isolates.

Finally, hydrogen sulfide production due to the activity of sulfite reductase was remarkably high in some *H. uvarum* and in most *H. osmophila* and *H. opuntiae* isolates, confirming a genus-related behavior. Regarding the other yeast species, only *S. cerevisiae* and *T. delbrueckii* had certain H_2_S-producer strains.

Thus, Figure S1 shows an active cluster at the lower region of the phylogenetic tree composed by basidiomycetous species (*C. amylolentus* and *R. toruloides*) and by *Metschnikowia* sp. and *A. pullulans* isolates. A highly inactive cluster in enzymatic terms could also be observed in the lower-middle zone.

### Origin-dependent intraspecific study

**Figure 4:**
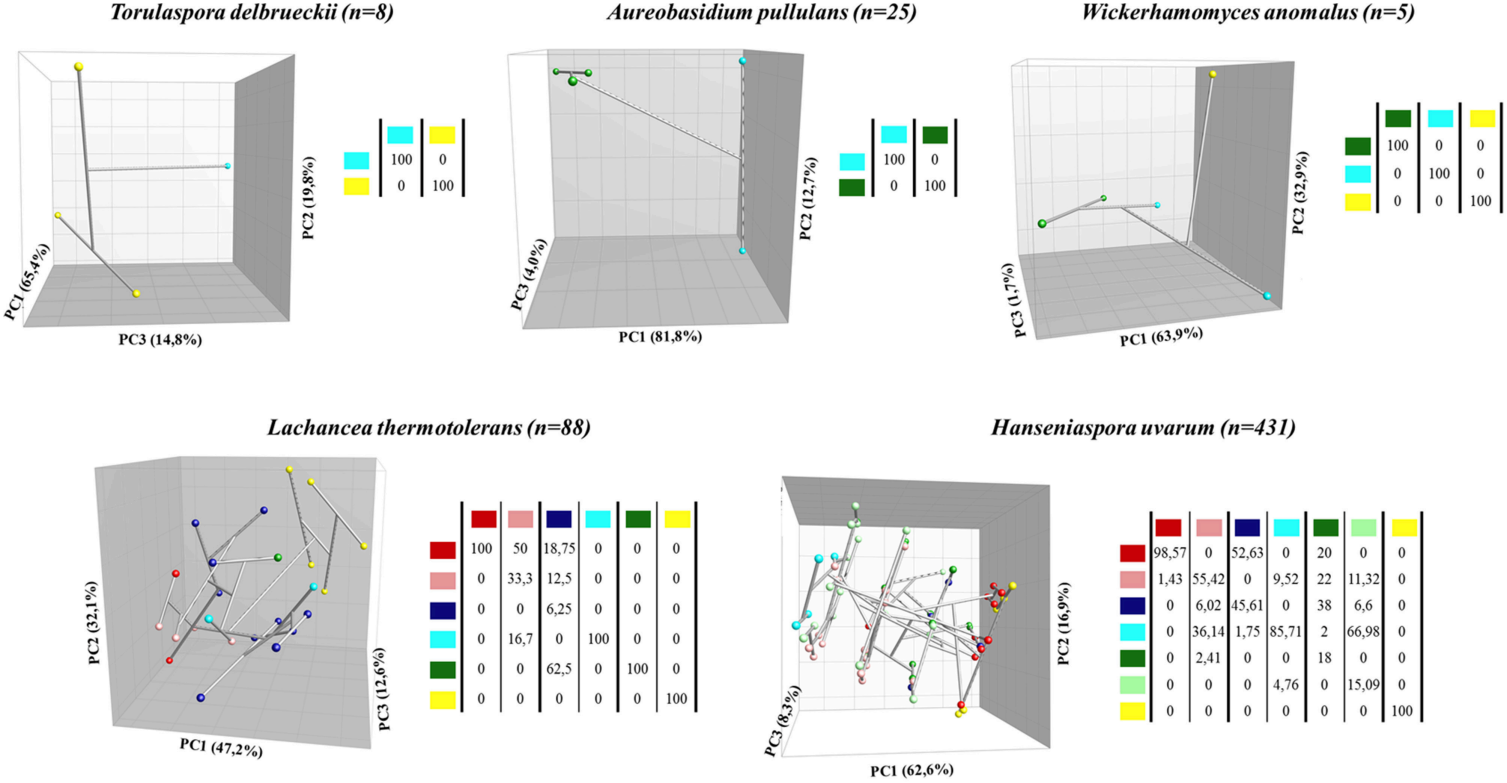
**Intraspecific distribution of isolates from the four origins and their corresponding vintages sampled**. Tridimensional plots correspond to the PCA analysis of specific populations considering their enzymatic activities, and group separation was calculated with the Jackknife method. Color legends: red (EM 2013), pink (EM 2014), blue (PDC 2013), cyan (PDC 2014), dark green (O 2013), pale green (O 2014), and yellow (G 2012). Tridimensional visualization was captured in order to optimize group distinction.

In order to study the concept of microbial terroir in depth, an intraspecific analysis was conducted on the enzymatic properties associated to every strain. Figure 4 shows the intraspecific clustering of the isolates of different species (five species isolated from more than one origin) by carrying out a PCA analysis using enzymatic data.

Considering the three less abundant species analyzed (*T. delbrueckii, A. pullulans*, and *W. anomalus*), it was possible to clearly establish origin-dependent strain clusters composed of homogeneous populations that could be distinguished by their enzymatic profiles. *T. delbrueckii* was isolated from the G (seven isolates) and PDC (one isolate) vineyards in the 2012 and 2014 vintages, respectively. Two different groups could be statistically identified (with two Principal Components (PCs) explaining 85.2% of the differences, and three PCs explaining 100%), showing a clear origin-dependent differentiation with β-glucosidase and pectinase mostly affecting this clustering (Figure 4, Table S2a). *A. pullulans* was also isolated from two vineyards: PDC (2014) and O (2013), with 20 and 5 isolates, respectively. In this case, two different groups were established depending on the isolation origin, composing 100% homogeneous population groups (Figure 4). The PCA analysis allowed us to statistically support this clustering, with the first two PCs explaining 94.55% of these differences, and three PCs explaining 98.51%. In this case, β-glucosidase and β-D-xylosidase were the factors mostly responsible for affecting this clustering, by greatly contributing to the first PC, which alone explains 81.84% of the established differences (Table S2b). *W. anomalus* was isolated from three different vineyards: G (2012), PDC (2014), and O (2013), with 1, 2 and 2 isolates, respectively, and these five isolates again described a phenotypic cluster according to their origin, composing three different phylo-functional groups (Figure 4). This clustering was again statistically significant in the PCA analysis, explaining 96.8% of the differences with the first two PCs, and 97.5% with three PCs. Protease activity was the most responsible factor, explaining the origin-dependent cluster separation, and contributing significantly to the first PC, which could explain 63.88% of the differences detected (Table S2c).

Due to their large sample size, the other two species evaluated (*L. thermotolerans* and *H. uvarum*) generate more complex clustering but, in most cases, some statistically homogeneous groups could be composed depending on the origin-dependent strain phenotype. Regarding *L. thermotolerans*, a total of 88 isolates were analyzed from G (2012), PDC (2013, 2014), EM (2013, 2014), and O (2013), with 6, 50, 31 and 1 isolates, respectively.

Clusters were established for the isolates from the four different vineyards, although a less precise separation could be established between the isolates of different years from the same vineyard. Figure 4 shows that *L. thermotolerans* isolates from EM (2013), PDC (2014), O (2013), and G (2012) established statistically homogeneous groups, defining their own enzymatic profile. Isolates from EM (2014) did not form a homogeneous group, but 50% of these isolates could be assigned to the EM (2013) enzymatic profile. Regarding PDC (2013) isolates, it was not possible to establish a uniform profile, with most of its isolates being similar to the enzymatic profiles from other origins. Apart from that, the PCA of the enzymatic properties of the total *L. thermotolerans* population could explain 79.28% of the differences between origins, considering the first two PCs, and 91.87% considering the first three PCs. These differences could be attributed mostly to β-D-xylosidase activity, H_2_S production, and β-glucosidase activity (Table S2d). Finally, regarding the largest species population in this study, the analysis of *H. uvarum* enzymatic profile generated the most complex clustering, although in some cases an origin-dependent enzymatic profile could be defined. *H. uvarum* was isolated from all the vineyards, reaching a total of 431 isolates from all sampled origins. Three origins established consistent groups: EM (2013), PDC (2014), and G (2012). On the other hand, *H. uvarum* isolates from O (2013 and 2014) did not establish a consistent enzymatic profile of their own, with most of the isolates being statistically attributed to other origin profiles. Finally, in an intermediate situation, EM (2014) and PDC (2013) originated not-fully consistent groups, with their enzymatic profile overlapping with the profile described by other vineyards from the same appellation (EM 2014 with PDC 2014; PDC 2013 with EM 2013) (Figure 4), describing a wider origin-specific profile. The PCA analysis of these data gives us statistical evidence of the significance of these clustering results. Sulfite reductase and β-D-xylosidase activities contributed notably to these differences, significantly affecting PC1, which could alone explain 62.62% of the differences between groups, and also PC2, which accumulates an explanation of 79.48% of the differences (Figure 4, Table S2e).

## Discussion

### Diversity and richness of yeast species

The main aim of this work was to establish a large collection of non-*Saccharomyces* yeasts isolated from different Spanish wine appellations in order to perform a joint phylo-functional analysis, linking phylogenetic and phenotypic data on the enzymatic properties of the yeast species identified. Furthermore, an attempt has been made to relate certain enzymatic activities, which are usually associated with certain yeasts, to the potential role they could play in enology.

The experimental approach developed for this study was based on culture-dependent techniques in order to obtain a yeast collection of enological origin that may have a use in winemaking. From a general point of view, our population data (Figure 1) were in line with other studies reporting that, apart from the *Aureobasidium* and *Rhodotorula* species that were intentionally avoided in this study as described in the yeast isolation procedure, *Hanseniaspora* spp., *Metschnikowia* spp., and *L. thermotolerans* dominate yeast communities in fresh musts (Prakitchaiwattana et al., 2004; Pinto et al., 2015), with *H. uvarum* accounting for more than half of the total yeast population isolated (Beltran et al., 2002; Wang et al., 2015).

There has recently been confirmation of the major differences in population richness values between culture-dependent and independent approaches in enological environments (Wang et al., 2015). Our overall results of yeast diversity using a culture-dependent approach are wider than those obtained in other similar studies. Wang et al. (2015) have managed to identify a total of three species (*H. uvarum, Issatchenkia terricola*, and *Starmerella bacillaris*) from a collection of 179 yeasts isolated from nine different origins by using a lysine medium, and five species (the three previously mentioned, together with *S. cerevisiae* and *Hanseniaspora valbyensis*) in 183 isolates from the same nine samples using YPD plates. The higher diversity obtained in our work (15 vs. 5 species) could be explained by both the larger sample size (770 vs. 362 isolates) and the greater heterogeneity in sampling areas (Figure 1). According to data reported by Beltran et al. (2002), several differences in yeast diversity were observed between years, as shown in Figure 2. Differences in the microbial composition among vintages, grape varieties, climate and location have been widely reported by Bokulich et al. (2014), and could account for the differences observed for yeast diversity found in the G vineyard compared to the diversity found in the other three vineyards studied (Figure S2, Table S3). The different microclimatic conditions, vineyard location and vine variety of this vineyard, with only the 2012 vintage sampled, could account for such a difference. The 2012 vintage in most Spanish wine appellations was characterized by low rainfall (Figure S2), which could restrict the filamentous fungi overgrowth that could displace some of the yeast species present in the grape microbial consortia (Liu et al., 2015). Additionally, as we show in this work, not only were the diversity and richness of yeast species affected by location, but also the phenotypic profile of the same yeast species differed across vineyards, and even in consecutive vintages (Figure 4).

Although most of the current population studies using culture-independent molecular methods report higher diversity values for fresh must than those reported here (Bokulich and Mills, 2013; David et al., 2014; Pinto et al., 2015), a wide variety of yeast species of enological interest (Jolly et al., 2014) were represented in the yeast collection established for their enzymatic characterization.

### Enzyme abundance and species-specific distribution

Regarding enzymatic screening, eight enzymatic activities were evaluated to establish an enzymatic profile of enological interest for the 15 yeast species studied (Figure 3). A group of three glycosidases (β-glucosidase, β-D-xylosidase, and α-L-arabinofuranosidase) were determined, recording different performances in terms of activity, distribution and abundance. According to other works (Fia et al., 2005), β-glucosidase was a widespread activity among wine yeasts. Our results have highlighted the β-glucosidase production of *Hanseniaspora* species, as well as of *M. guilliermondii* and *W. anomalus*. These results are also consistent with other enzymatic screenings that additionally reported the ability of some *H. uvarum* strains to produce versatile β-glucosidase enzymes with no repression by glucose and with no significant activity decrease in a wide range of pH values (López et al., 2015). Delcroix et al. (1994) and Hernández et al. (2002) evidenced a loss of stability of β-glucosidase in *S. cerevisiae*, with a strong reduction in its enzymatic activity (about 80%) when changing from pH 5 to pH 3, while other authors have reported a notable decrease in most non-*Saccharomyces* species at pH values below 4 (Rosi et al., 1994). However, Mateo et al. (2011) have reported that *W. anomalus* reached its maximum β-glucosidase activity at pH 3.2, also recording lower rates of catabolic repression by glucose. Thus, with β-glucosidase being the final activity responsible for the release of wine terpenes from their glycosylated precursors, both *Hanseniaspora* species and *W. anomalus* seem to be a useful tool to increase wine terpenics, as suggested by Mendes-Ferreira et al. (2001) and Mateo et al. (2011), respectively.

Regarding the other two glycosidases analyzed (β-D-xylosidase and α-L-arabinofuranosidase), different abundances were observed among the yeast population studied. Contrary to what was observed in β-glucosidase activity, *Hanseniaspora* spp. had neither β-D-xylosidase (with the exception of *H. osmophila* and a few *H. uvarum* strains) nor α-L-arabinofuranosidase activities, which was in complete agreement with previous observations reported by Manzanares et al. (1999). However, they also highlighted a remarkable β-D-xylosidase activity for some *W. anomalus* and *H. uvarum* strains at the usual enological pH values of 3–3.8, with their use also being recommended for terpene release in wine fermentation. Furthermore, lower repression levels by glucose and ethanol have been reported for *W. anomalus* glycosidase activities (Mateo et al., 2011). Regarding the other yeast isolates, a β-D-xylosidase-active cluster was observed in the phylogenetically related species *T. delbrueckii, Z. bailii*, and *S. cerevisiae*. However, a high glucose-dependent repression has been observed in these species (Gueguen et al., 1995; Mateo and Di Stefano, 1997; Mateo et al., 2011), restricting their use in terpene release in wine fermentation.

Finally, α-L-arabinofuranosidase, as the least distributed glycosidase, was observed in *M. guilliermondii, W. anomalus, A. pullulans, R. toruloides*, and *C. amylolentus*. McMahon et al. (1999) have reported the major ability *A. pullulans* glycosidases have to release wine terpene glycosides. According to Mateo et al. (2011), α-L-arabinofuranosidase, together with α-L-rhamnosidase, is the least glucose-repressed glycosidase in wine yeasts, so both are of enological interest. Regarding *Metschnikowia* spp., most of them had remarkable β-glucosidase and β-D-xylosidase activities, although a considerable number of *Metschnikowia* sp. (not considering *M. viticola* isolates) had also α-L-arabinofuranosidase activity. Along these lines, it has been reported that a commercial strain of *M. pulcherrima* could increase volatile terpenes in wine due to its α-L-arabinofuranosidase activity (Lallemand, 2013).

Overall, our results are in agreement with other works reporting that *Pichia, Wickerhamomyces*, and *Hanseniaspora* genera are major producers of glycosidase enzymes (Manzanares et al., 1999) and, furthermore, we report the remarkable glycosidase activity of wine-related basidiomycetes, such as *R. toruloides* and *C. amylolentus*.

β-Lyase activity, which is also directly related to varietal aroma enhancement, recorded a moderate distribution in the overall yeast collection studied. Figure 3 shows moderate β-lyase activity in the majority of yeast species, with its production being remarkable in *T. delbrueckii, K. marxianus*, and *M. guilliermondii*. Although this activity has been studied in depth in *S. cerevisiae* wine strains (Howell et al., 2005; Thibon et al., 2008; Roncoroni et al., 2011), actual information on the ability of non-*Saccharomyces* to release volatile thiols in wine is very scarce. Zott et al. (2011) have reported that β-lyase activity is a strain-dependent characteristic in non-*Saccharomyces* yeasts, as described in *S. cerevisiae* (Roncoroni et al., 2011; Santiago and Gardner, 2015). Accordingly, Figure 3 shows that β-lyase activity has great intraspecific variability. Zott et al. (2011) have reported that, apart from *T. delbrueckii*, some *M. pulcherrima* and *L. thermotolerans* strains have the ability to release volatile thiols in Sauvignon Blanc wines, but only a few strains of these species have recorded β-lyase activity in our *in vitro* assays. Regarding the *Hanseniaspora* genus, and as occurred with β-D-xylosidase, *H. osmophila* recorded higher β-lyase activity compared to *H. opuntiae* and *H. uvarum* species. These phenotypical differences were consistent with the observations made in the phylogenetic tree (Figure S1), in which *H. osmophila* was distant from the *Hanseniaspora* genus cluster. Due to the high nitrogen catabolic repression affecting β-lyase activity in *S. cerevisiae*, which restricts its contribution to thiol release in wine fermentation (Thibon et al., 2008), these alternative yeasts should be studied in depth as a way to improve volatile thiol release in enological conditions.

H_2_S production, as a result of sulfite reductase activity, is a rare feature among the majority of non-*Saccharomyces* species. Furthermore, as occurred with β-lyase (the other sulfur-related activity), major intraspecific variability could be observed in species such as *H. uvarum* and *L. thermotolerans*, as well as in *S. cerevisiae*, as previously reported by Linderholm et al. (2008). Given that the nitrogen composition of musts has been described to affect H_2_S production by yeasts (Linderholm et al., 2008), and since non-*Saccharomyces* yeasts have high nutritional demands (Jolly et al., 2014), the lack of sulfite reductase activity in most of them is a positive characteristic for their application without the risk of wine reduction.

Protease, pectinase and cellulase have been studied for their involvement in several technological processes in winemaking. Figure 3 shows that protease is a widespread activity when the total population of yeasts is considered, in agreement with previous works (Lagace and Bisson, 1990; Chomsri, 2008). This is caused by the protease activity of the most abundant species (*Hanseniaspora* species and *Metschnikowia* sp.), although other species of enological interest with a lower relative abundance recorded no activity (*S. cerevisiae, T. delbrueckii*, and *L. thermotolerans*, among others). In addition, protease and pectinase seem to be the main differential activities between *M. viticola* and the other *Metschnikowia* species isolated. The use of proteases in winemaking is not a widely extended practice at the moment, with bentonite being used more often to solve protein haze problems. The use of bentonite usually impairs the sensorial properties of wines, so the use of proteases for this purpose seems to be a potential future application (Marangon et al., 2012). Special note should be taken of the high protease activity of *W. anomalus*, especially in the NS-PDC-167 strain (Figure 3, Figure S1), which should be studied in depth for its application in protein haze prevention. In fact, an aspartate-protease from *M. pulcherrima* has been characterized and expressed in *S. cerevisiae* by Reid et al. (2012) for its potential wine application, but the role of proteases from yeasts in winemaking is still poorly understood.

Regarding pectinolytic activity, different studies have confirmed that most yeast species are unable to produce pectic enzymes. It should be mentioned that polygalacturonase activity has been reported in a few wine yeast isolates without establishing a species-specific behavior (Strauss et al., 2001; Merín et al., 2011). In this context, our results suggest that *M. pulcherrima, M. fructicola* (jointly identified here as *Metschnikowia* sp.), and *A. pullulans* are leading candidates for their use as a source of pectinase in winemaking. Following the confirmed usefulness of pectinases from *A. pullulans* in winemaking conditions (Merín and Morata de Ambrosini, 2015), the impact of *M. pulcherrima*, improving phenolic extraction and clarification processes in some pectinase-dependent wine properties, has recently been confirmed (Belda et al., unpublished). Furthermore, in light of the behavior of *A. pullulans*, this was the only cellulase-active species in the collection studied, in contrast with data reported by Strauss et al. (2001) and Merín et al. (2015) which describe the presence of cellulase activity in some ascomycetous yeasts (*Candida stellata, M. pulcherrima*, and *H. uvarum*) and in the basidiomycetous yeast *Rhodotorula dairenensis*, respectively.

It has been reported that at least 75% of the *S. cerevisiae* enological strains have limited pectinolytic activity (Blanco et al., 1994). However, Merín et al. (2011) and Merín and Morata de Ambrosini (2015) have confirmed the existence of a constitutive pectinase activity not repressed by glucose in non-*Saccharomyces* species, in contrast with what occurred in *S. cerevisiae* (Radoi et al., 2005). In this context, our results confirm that the vast majority of *Metschnikowia* sp. and *A. pullulans* strains are of interest for their use as pectinase sources in enology, opening a new research line for their industrial application.

### Origin-dependent intraspecific phenotypic profiles

Metagenomic approaches have allowed researchers to definitively establish the concept of microbial terroir, relating location and climatic factors to specific microbial populations in vineyards (Bokulich et al., 2014). This finding has been put forward as a determinant in the differential flavor and aroma profiles of wines from different origins (Gilbert et al., 2014). Additionally, our results confirm that significant phenotypical differences could be observed between strains of the same species from different origins, delving further into the concept of microbial terroir, for the first time at strain level.

The results shown in Figure 4 allow us to confirm the possibility of separating single species populations based on their enzymatic properties establishing origin-dependent clusters. It has been suggested that high-throughput screening (HTS) assays are crucial for discovering interesting enzymes and new sources (Goddard and Reymond, 2004). Here, we also report the potential these techniques have to develop phylo-functional analyses of yeast communities to perform innovative ecological studies. A similar approach has recently been adopted by Zhang et al. (2015) to establish phylo-functional differences among the gut microbiota of different human populations.

The connecting lines shown in Figure 4 have allowed us to decipher the phylogenetic relationships among groups of isolates according to their phenotypical similarities. The tridimensional plot for *T. delbrueckii, A. pullulans*, and *W. anomalus* shows highly defined origin-dependent clusters with significant percentages of statistical differences among groups, bearing in mind that they were scarcely isolated. The population distribution of *L. thermotolerans* and *H. uvarum* isolates shown in the tridimensional plot could be better interpreted considering numerical data for group homogeneity (Figure 4) because of the high number of isolates considered. The results for both species isolated from Ribera del Duero vineyards (EM and PDC) suggest that the EM population isolated in 2014 was more heterogeneous when compared with data for 2013. In contrast, yeast populations from the PDC vineyard followed the opposite trend, with the populations being more homogeneous in 2014 for both species, as compared to 2013. These differences, together with the different behavior of EM and PDC populations shown in Figure 2, could be related to microclimatic determinants and to viticulture practices conditioning the health status of grapes that could determine microbial populations in them (Sipiczki, 2006; Barata et al., 2008). In the case of *H. uvarum* isolates from the O vineyard (Rueda wine appellation), the populations obtained in both the 2013 and 2014 vintages were very heterogeneous. As they were the only species analyzed for consecutive vintages in this vineyard, it is not possible to draw a wider conclusion about the intraspecific consistency in the O vineyard. It may be the case that the biodynamic practices applied in this vineyard contribute to a great microbial diversity, as suggested by Setati et al. (2012). The wide gap between the G population and the other population groups could be explained by geographic and climatic reasons, as it has been isolated in a wine appellation (Tierra de León) with several climatic and orographic differences with respect to its Ribera del Duero and Rueda counterparts, as well as in a different vintage (2012) with certain weather peculiarities (remarkably low rainfall).

In summary, the phenotypical characterization of our yeast population goes deep into the concept of microbial terroir, considering the yeast diversity at strain level as an important factor for determining the microbial influence on the flavor properties of wines. This intraspecific phenotypical clustering could not have been explored with current metagenomic approaches. However, the exponential growth of genomic data for non-*Saccharomyces* species and the versatility of high throughput genomic techniques, together with data on the species-specific enzymatic profiles reported in this work, open new possibilities for future comparative genomic works that will allow for the targeted development of high throughput metabolic screenings.

## Author contributions

AS, EN, and DM conceived the project; IB, AS, EN, and JR designed and performed the experiments; IB, AA, and AS analyzed the data, and IB and AS wrote and edited the manuscript.

### Conflict of interest statement

The authors declare that the research was conducted in the absence of any commercial or financial relationships that could be construed as a potential conflict of interest.
