# Supplementary material for: Unraveling the Enzymatic Basis of Wine “Flavorome”: A Phylo-Functional Study of Wine Related Yeast Species
Source: Front Microbiol. 2016 Jan 20;7:12. doi: 10.3389/fmicb.2016.00012 (PMC4718978; doi:10.3389/fmicb.2016.00012)
Supplement: Supplementary file 1 [file Table1.PDF]

Supplementary material. Belda et al.
Unraveling the enzymatic basis of wine "flavorome": a phylo-functional study of wine related yeast species

Table S1: Yeast collection with genbank accession numbers

	ISOLATES IDENTIFICATION		
Origin	Strain code	Genbank accession number	Identification (species)
	NS-O-1	KT922724	*Hanseniaspora uvarum*
	NS-O-2	KT922725	*Hanseniaspora uvarum*
	NS-O-3	KT922726	*Hanseniaspora uvarum*
	NS-O-4	KT922727	*Hanseniaspora uvarum*
	NS-O-5	KT922728	*Hanseniaspora uvarum*
	NS-O-6	KT922729	*Hanseniaspora uvarum*
	NS-O-7	KT922730	*Hanseniaspora uvarum*
	NS-O-8	KT922731	*Hanseniaspora uvarum*
	NS-O-9	KT922732	*Hanseniaspora uvarum*
	NS-O-10	KT922733	*Hanseniaspora uvarum*
	NS-O-11	KT922734	*Wickerhamomyces anomalus*
	NS-O-12	KT922735	*Hanseniaspora uvarum*
	NS-O-13	KT922736	*Hanseniaspora uvarum*
	NS-O-14	KT922737	*Wickerhamomyces anomalus*
	NS-O-15	KT922738	*Hanseniaspora uvarum*
	NS-O-16	KT922739	*Hanseniaspora uvarum*
	NS-O-17	KT922740	*Hanseniaspora uvarum*
	NS-O-18	KT922741	*Hanseniaspora uvarum*
	NS-O-19	KT922742	*Hanseniaspora uvarum*
	NS-O-20	KT922743	*Hanseniaspora uvarum*
	NS-O-21	KT922744	*Hanseniaspora uvarum*
	NS-O-22	KT922745	*Hanseniaspora uvarum*
	NS-O-23	KT922746	*Hanseniaspora uvarum*
	NS-O-24	KT922747	*Hanseniaspora osmophila*
	NS-O-25	KT922748	*Hanseniaspora uvarum*
	NS-O-26	KT922749	*Hanseniaspora uvarum*
	NS-O-27	KT922750	*Hanseniaspora uvarum*
	NS-O-28	KT922751	*Hanseniaspora uvarum*
	NS-O-29	KT922752	*Metschnikowia sp.*
	NS-O-30	KT922753	*Hanseniaspora uvarum*
	NS-O-31	KT922754	*Hanseniaspora uvarum*
	NS-O-32	KT922755	*Metschnikowia viticola*
	NS-O-33	KT922756	*Metschnikowia sp.*
	NS-O-34	KT922757	*Hanseniaspora uvarum*
	NS-O-35	KT922758	*Metschnikowia viticola*
	NS-O-36	KT922759	*Metschnikowia viticola*
	NS-O-37	KT922760	*Metschnikowia sp.*
	NS-O-38	KT922761	*Hanseniaspora uvarum*
	NS-O-39	KT922762	*Hanseniaspora uvarum*

O (2013)	NS-O-40	KT922763	*Hanseniaspora uvarum*
	NS-O-41	KT922764	*Hanseniaspora uvarum*
	NS-O-42	KT922765	*Hanseniaspora uvarum*
	NS-O-43	KT922766	*Hanseniaspora uvarum*
	NS-O-44	KT922767	*Hanseniaspora uvarum*
	NS-O-45	KT922768	*Metschnikowia viticola*
	NS-O-46	KT922769	*Lachancea thermotolerans*
	NS-O-47	KT922770	*Hanseniaspora uvarum*
	NS-O-48	KT922771	*Hanseniaspora uvarum*
	NS-O-49	KT922772	*Hanseniaspora uvarum*
	NS-O-50	KT922773	*Hanseniaspora uvarum*
	NS-O-51	KT922774	*Hanseniaspora uvarum*
	NS-O-52	KT922775	*Hanseniaspora uvarum*
	NS-O-53	KT922776	*Hanseniaspora uvarum*
	NS-O-54	KT922777	*Hanseniaspora uvarum*
	NS-O-55	KT922778	*Hanseniaspora uvarum*
	NS-O-56	KT922779	*Hanseniaspora uvarum*
	NS-O-57	KT922780	*Hanseniaspora uvarum*
	NS-O-58	KT922781	*Hanseniaspora uvarum*
	NS-O-59	KT922782	*Hanseniaspora uvarum*
	NS-O-60	KT922783	*Hanseniaspora uvarum*
	NS-O-61	KT922784	*Metschnikowia sp.*
	NS-O-62	KT922785	*Metschnikowia sp.*
	NS-O-63	KT922786	*Metschnikowia sp.*
	NS-O-64	KT922787	*Metschnikowia sp.*
	NS-O-65	KT922788	*Metschnikowia sp.*
	NS-O-66	KT922789	*Metschnikowia sp.*
	NS-O-67	KT922790	*Metschnikowia sp.*
	NS-O-68	KT922791	*Metschnikowia sp.*
	NS-O-69	KT922792	*Metschnikowia sp.*
	NS-O-70	KT922793	*Aureobasidium pullulans*
	NS-O-71	KT922794	*Metschnikowia sp.*
	NS-O-73	KT922795	*Aureobasidium pullulans*
	NS-O-74	KT922796	*Hanseniaspora uvarum*
	NS-O-75	KT922797	*Metschnikowia sp.*
	NS-O-76	KT922798	*Metschnikowia sp.*
	NS-O-77	KT922799	*Metschnikowia sp.*
	NS-O-78	KT922800	*Metschnikowia sp.*
	NS-O-79	KT922801	*Metschnikowia sp.*
	NS-O-80	KT922802	*Metschnikowia sp.*
	NS-O-81	KT922803	*Metschnikowia sp.*
	NS-O-82	KT222663	*Aureobasidium pullulans*
	NS-O-83	KT922804	*Metschnikowia sp.*
	NS-O-84	KT922805	*Metschnikowia sp.*
	NS-O-85	KT922806	*Metschnikowia sp.*
	NS-O-86	KT922807	*Metschnikowia sp.*

	NS-O-87	KT922808	*Metschnikowia sp.*
	NS-O-88	KT922809	*Metschnikowia sp.*
	NS-O-89	KT922810	*Metschnikowia sp.*
	NS-O-90	KT922811	*Metschnikowia sp.*
	NS-O-91	KT922812	*Metschnikowia sp.*
	NS-O-92	KT922813	*Metschnikowia sp.*
	NS-O-93	KT922814	*Metschnikowia sp.*
	NS-O-94	KT922815	*Metschnikowia viticola*
	NS-O-97	KT922816	*Metschnikowia viticola*
	NS-O-98	KT922817	*Metschnikowia viticola*
	NS-O-99	KT922818	*Metschnikowia sp.*
	NS-O-100	KT922819	*Metschnikowia viticola*
	NS-O-101	KT922820	*Metschnikowia sp.*
	NS-O-102	KT922821	*Metschnikowia viticola*
	NS-O-103	KT922822	*Metschnikowia sp.*
	NS-O-104	KT922823	*Metschnikowia sp.*
	NS-O-105	KT922824	*Aureobasidium pullulans*
	NS-O-106	KT922825	*Metschnikowia sp.*
	NS-O-107	KT922826	*Metschnikowia viticola*
	NS-O-108	KT922827	*Metschnikowia viticola*
	NS-O-109	KT922828	*Aureobasidium pullulans*
	NS-O-110	KT922829	*Metschnikowia viticola*
	NS-O-111	KT922830	*Metschnikowia viticola*
	NS-O-112	KT922831	*Metschnikowia viticola*
	NS-O-113	KT922832	*Metschnikowia viticola*
	NS-O-114	KT922833	*Metschnikowia viticola*
	NS-O-115	KT922834	*Metschnikowia viticola*
	NS-O-116	KT922835	*Metschnikowia viticola*
	NS-O-117	KT922836	*Metschnikowia viticola*
	NS-O-118	KT922837	*Metschnikowia viticola*
	NS-O-119	KT922838	*Metschnikowia viticola*
	NS-O-120	KT922839	*Hanseniaspora uvarum*
	NS-O-121	KT922840	*Hanseniaspora uvarum*
	NS-O-122	KT922841	*Hanseniaspora uvarum*
	NS-O-123	KT922842	*Hanseniaspora uvarum*
	NS-O-124	KT922843	*Hanseniaspora uvarum*
	NS-O-125	KT922844	*Hanseniaspora uvarum*
	NS-O-126	KT922845	*Hanseniaspora uvarum*
	NS-O-127	KT922846	*Hanseniaspora uvarum*
	NS-O-128	KT922847	*Hanseniaspora uvarum*
	NS-O-129	KT922848	*Hanseniaspora uvarum*
	NS-O-130	KT922849	*Hanseniaspora uvarum*
	NS-O-131	KT922850	*Hanseniaspora uvarum*
	NS-O-132	KT922851	*Hanseniaspora uvarum*
	NS-O-133	KT922852	*Hanseniaspora uvarum*
	NS-O-134	KT922853	*Hanseniaspora uvarum*

	NS-O-135	KT922854	*Hanseniaspora uvarum*
	NS-O-136	KT922855	*Hanseniaspora uvarum*
	NS-O-137	KT922856	*Hanseniaspora uvarum*
	NS-O-138	KT922857	*Hanseniaspora uvarum*
	NS-O-139	KT922858	*Hanseniaspora uvarum*
	NS-O-140	KT922859	*Hanseniaspora uvarum*
	NS-O-141	KT922860	*Hanseniaspora uvarum*
	NS-O-142	KT922861	*Metschnikowia sp.*
	NS-O-143	KT922862	*Hanseniaspora uvarum*
	NS-O-144	KT922863	*Hanseniaspora uvarum*
	NS-O-145	KT922864	*Hanseniaspora uvarum*
	NS-O-146	KT922865	*Hanseniaspora uvarum*
	NS-O-147	KT922866	*Hanseniaspora uvarum*
	NS-O-148	KT922867	*Hanseniaspora uvarum*
	NS-O-149	KT922868	*Hanseniaspora uvarum*
	NS-O-150	KT922869	*Hanseniaspora uvarum*
	NS-O-151	KT922870	*Hanseniaspora uvarum*
	NS-O-152	KT922871	*Hanseniaspora uvarum*
	NS-O-153	KT922872	*Hanseniaspora uvarum*
	NS-O-154	KT922873	*Hanseniaspora uvarum*
	NS-O-155	KT922874	*Hanseniaspora uvarum*
	NS-O-156	KT922875	*Hanseniaspora uvarum*
	NS-O-157	KT922876	*Hanseniaspora uvarum*
	NS-O-158	KT922877	*Hanseniaspora uvarum*
	NS-O-159	KT922878	*Hanseniaspora uvarum*
	NS-O-160	KT922879	*Hanseniaspora uvarum*
	NS-O-161	KT922880	*Hanseniaspora uvarum*
	NS-O-162	KT922881	*Hanseniaspora uvarum*
	NS-O-163	KT922882	*Hanseniaspora uvarum*
	NS-O-164	KT922883	*Hanseniaspora uvarum*
	NS-O-165	KT922884	*Hanseniaspora uvarum*
	NS-O-166	KT922885	*Hanseniaspora uvarum*
	NS-O-167	KT922886	*Hanseniaspora uvarum*
	NS-O-168	KT922887	*Hanseniaspora uvarum*
	NS-O-169	KT922888	*Hanseniaspora uvarum*
	NS-O-170	KT922889	*Hanseniaspora uvarum*
	NS-O-171	KT922890	*Hanseniaspora uvarum*
	NS-O-172	KT922891	*Hanseniaspora uvarum*
	NS-O-173	KT922892	*Hanseniaspora uvarum*
	NS-O-174	KT922893	*Hanseniaspora uvarum*
	NS-O-175	KT922894	*Hanseniaspora uvarum*
	NS-O-176	KT922895	*Hanseniaspora uvarum*
	NS-O-177	KT922896	*Hanseniaspora uvarum*
	NS-O-178	KT922897	*Hanseniaspora uvarum*
	NS-O-179	KT922898	*Hanseniaspora uvarum*
	NS-O-180	KT922899	*Hanseniaspora uvarum*

O (2014)	NS-O-181	KT922900	*Hanseniaspora uvarum*
	NS-O-182	KT922901	*Hanseniaspora uvarum*
	NS-O-183	KT922902	*Hanseniaspora uvarum*
	NS-O-184	KT922903	*Hanseniaspora uvarum*
	NS-O-185	KT922904	*Hanseniaspora uvarum*
	NS-O-186	KT922905	*Hanseniaspora uvarum*
	NS-O-187	KT922906	*Hanseniaspora uvarum*
	NS-O-188	KT922907	*Hanseniaspora uvarum*
	NS-O-189	KT922908	*Hanseniaspora uvarum*
	NS-O-190	KT922909	*Hanseniaspora uvarum*
	NS-O-191	KT922910	*Hanseniaspora uvarum*
	NS-O-192	KT922911	*Hanseniaspora uvarum*
	NS-O-193	KT922912	*Hanseniaspora uvarum*
	NS-O-194	KT922913	*Hanseniaspora uvarum*
	NS-O-195	KT922914	*Hanseniaspora uvarum*
	NS-O-196	KT922915	*Hanseniaspora uvarum*
	NS-O-197	KT922916	*Hanseniaspora uvarum*
	NS-O-198	KT922917	*Hanseniaspora uvarum*
	NS-O-199	KT922918	*Hanseniaspora uvarum*
	NS-O-200	KT922919	*Hanseniaspora uvarum*
	NS-O-201	KT922920	*Hanseniaspora uvarum*
	NS-O-202	KT922921	*Hanseniaspora uvarum*
	NS-O-203	KT922922	*Hanseniaspora uvarum*
	NS-O-204	KT922923	*Hanseniaspora uvarum*
	NS-O-205	KT922924	*Hanseniaspora uvarum*
	NS-O-206	KT922925	*Hanseniaspora uvarum*
	NS-O-207	KT922926	*Hanseniaspora uvarum*
	NS-O-208	KT922927	*Hanseniaspora uvarum*
	NS-O-209	KT922928	*Hanseniaspora uvarum*
	NS-O-210	KT922929	*Hanseniaspora uvarum*
	NS-O-211	KT922930	*Hanseniaspora uvarum*
	NS-O-212	KT922931	*Hanseniaspora uvarum*
	NS-O-213	KT922932	*Hanseniaspora uvarum*
	NS-O-214	KT922933	*Hanseniaspora uvarum*
	NS-O-215	KT922934	*Hanseniaspora uvarum*
	NS-O-216	KT922935	*Hanseniaspora uvarum*
	NS-O-217	KT922936	*Hanseniaspora uvarum*
	NS-O-218	KT922937	*Hanseniaspora uvarum*
	NS-O-219	KT922938	*Hanseniaspora uvarum*
	NS-O-220	KT922939	*Hanseniaspora uvarum*
	NS-O-221	KT922940	*Metschnikowia sp.*
	NS-O-222	KT922941	*Metschnikowia sp.*
	NS-O-223	KT922942	*Metschnikowia sp.*
	NS-O-224	KT922943	*Metschnikowia sp.*
	NS-O-225	KT922944	*Metschnikowia sp.*
	NS-O-226	KT922945	*Metschnikowia sp.*

	NS-O-227	KT922946	*Metschnikowia sp.*
	NS-O-228	KT922947	*Metschnikowia sp.*
	NS-O-229	KT922948	*Metschnikowia sp.*
	NS-O-230	KT922949	*Metschnikowia sp.*
	NS-O-231	KT922950	*Metschnikowia sp.*
	NS-O-232	KT922951	*Metschnikowia sp.*
	NS-O-233	KT922952	*Metschnikowia sp.*
	NS-O-234	KT922953	*Metschnikowia sp.*
	NS-O-235	KT922954	*Metschnikowia sp.*
	NS-O-236	KT922955	*Metschnikowia sp.*
	NS-O-237	KT922956	*Metschnikowia sp.*
	NS-O-238	KT922957	*Metschnikowia sp.*
	NS-O-239	KT922958	*Metschnikowia sp.*
	NS-O-240	KT922959	*Metschnikowia sp.*
	NS-O-241	KT922960	*Hanseniaspora uvarum*
	NS-O-242	KT922961	*Hanseniaspora uvarum*
	NS-O-243	KT922962	*Hanseniaspora uvarum*
	NS-O-244	KT922963	*Metschnikowia sp.*
	NS-O-245	KT922964	*Hanseniaspora uvarum*
	NS-O-246	KT922965	*Hanseniaspora uvarum*
	NS-O-247	KT922966	*Hanseniaspora uvarum*
	NS-O-248	KT922967	*Metschnikowia sp.*
	NS-O-249	KT922968	*Metschnikowia sp.*
	NS-O-250	KT922969	*Hanseniaspora uvarum*
	NS-PDC-1	KT922471	*Hanseniaspora uvarum*
	NS-PDC-2	KT922472	*Hanseniaspora uvarum*
	NS-PDC-3	KT922473	*Hanseniaspora uvarum*
	NS-PDC-4	KT922474	*Hanseniaspora uvarum*
	NS-PDC-5	KT922475	*Hanseniaspora uvarum*
	NS-PDC-6	KT922476	*Hanseniaspora uvarum*
	NS-PDC-7	KT922477	*Hanseniaspora uvarum*
	NS-PDC-8	KT922478	*Hanseniaspora uvarum*
	NS-PDC-9	KT922479	*Hanseniaspora uvarum*
	NS-PDC-10	KT922480	*Hanseniaspora uvarum*
	NS-PDC-11	KT922481	*Hanseniaspora uvarum*
	NS-PDC-12	KT922482	*Hanseniaspora uvarum*
	NS-PDC-13	KT922483	*Hanseniaspora uvarum*
	NS-PDC-14	KT922484	*Metschnikowia sp.*
	NS-PDC-15	KT922485	*Hanseniaspora uvarum*
	NS-PDC-16	KT922486	*Hanseniaspora uvarum*
	NS-PDC-17	KT922487	*Hanseniaspora uvarum*
	NS-PDC-18	KT922488	*Hanseniaspora uvarum*
	NS-PDC-19	KT922489	*Hanseniaspora uvarum*
	NS-PDC-20	KT922490	*Hanseniaspora uvarum*
	NS-PDC-21	KT922491	*Hanseniaspora uvarum*
	NS-PDC-22	KT922492	*Hanseniaspora uvarum*

PDC (2013)	NS-PDC-23	KT922493	*Hanseniaspora uvarum*
	NS-PDC-24	KT922494	*Hanseniaspora uvarum*
	NS-PDC-25	KT922495	*Hanseniaspora uvarum*
	NS-PDC-26	KT922496	*Hanseniaspora uvarum*
	NS-PDC-27	KT922497	*Hanseniaspora uvarum*
	NS-PDC-28	KT922498	*Hanseniaspora uvarum*
	NS-PDC-29	KT922499	*Hanseniaspora uvarum*
	NS-PDC-30	KT922500	*Hanseniaspora uvarum*
	NS-PDC-31	KT922501	*Hanseniaspora uvarum*
	NS-PDC-32	KT922502	*Hanseniaspora uvarum*
	NS-PDC-33	KT922503	*Hanseniaspora uvarum*
	NS-PDC-34	KT922504	*Hanseniaspora uvarum*
	NS-PDC-35	KT922505	*Hanseniaspora uvarum*
	NS-PDC-36	KT922506	*Hanseniaspora uvarum*
	NS-PDC-37	KT922507	*Hanseniaspora uvarum*
	NS-PDC-38	KT922508	*Hanseniaspora uvarum*
	NS-PDC-39	KT886435	*Hanseniaspora uvarum*
	NS-PDC-40	KT922509	*Hanseniaspora uvarum*
	NS-PDC-41	KT922510	*Lachancea thermotolerans*
	NS-PDC-42	KT922511	*Lachancea thermotolerans*
	NS-PDC-43	KT922512	*Lachancea thermotolerans*
	NS-PDC-44	KT922513	*Lachancea thermotolerans*
	NS-PDC-45	KT922514	*Lachancea thermotolerans*
	NS-PDC-46	KT922515	*Lachancea thermotolerans*
	NS-PDC-47	KT922516	*Lachancea thermotolerans*
	NS-PDC-48	KT922517	*Metschnikowia sp.*
	NS-PDC-49	KT922518	*Lachancea thermotolerans*
	NS-PDC-50	KT922519	*Metschnikowia sp.*
	NS-PDC-51	KT922520	*Metschnikowia sp.*
	NS-PDC-52	KT922521	*Metschnikowia sp.*
	NS-PDC-53	KT922522	*Metschnikowia sp.*
	NS-PDC-54	KT922523	*Metschnikowia sp.*
	NS-PDC-55	KT922524	*Metschnikowia sp.*
	NS-PDC-56	KT922525	*Metschnikowia sp.*
	NS-PDC-57	KT922526	*Metschnikowia sp.*
	NS-PDC-58	KT922527	*Lachancea thermotolerans*
	NS-PDC-59	KT922528	*Lachancea thermotolerans*
	NS-PDC-60	KT922529	*Lachancea thermotolerans*
	NS-PDC-61	KT922530	*Lachancea thermotolerans*
	NS-PDC-62	KT922531	*Lachancea thermotolerans*
	NS-PDC-63	KT922532	*Lachancea thermotolerans*
	NS-PDC-64	KT922533	*Lachancea thermotolerans*
	NS-PDC-65	KT922534	*Lachancea thermotolerans*
	NS-PDC-66	KT922535	*Lachancea thermotolerans*
	NS-PDC-67	KT922536	*Lachancea thermotolerans*
	NS-PDC-68	KT922537	*Lachancea thermotolerans*

NS-PDC-69	KT922538	*Lachancea thermotolerans*
NS-PDC-70	KT922539	*Lachancea thermotolerans*
NS-PDC-71	KT922540	*Lachancea thermotolerans*
NS-PDC-72	KT922541	*Lachancea thermotolerans*
NS-PDC-73	KT922542	*Lachancea thermotolerans*
NS-PDC-74	KT922543	*Lachancea thermotolerans*
NS-PDC-75	KT922544	*Lachancea thermotolerans*
NS-PDC-76	KT922545	*Lachancea thermotolerans*
NS-PDC-77	KT922546	*Lachancea thermotolerans*
NS-PDC-78	KT922547	*Lachancea thermotolerans*
NS-PDC-79	KT922548	*Lachancea thermotolerans*
NS-PDC-80	KT922549	*Lachancea thermotolerans*
NS-PDC-81	KT922550	*Metschnikowia sp.*
NS-PDC-82	KT922551	*Lachancea thermotolerans*
NS-PDC-83	KT922552	*Lachancea thermotolerans*
NS-PDC-84	KT922553	*Lachancea thermotolerans*
NS-PDC-85	KT922554	*Lachancea thermotolerans*
NS-PDC-86	KT922555	*Lachancea thermotolerans*
NS-PDC-87	KT922556	*Lachancea thermotolerans*
NS-PDC-88	KT922557	*Lachancea thermotolerans*
NS-PDC-89	KT922558	*Lachancea thermotolerans*
NS-PDC-90	KT922559	*Lachancea thermotolerans*
NS-PDC-91	KT922560	*Lachancea thermotolerans*
NS-PDC-92	KT922561	*Lachancea thermotolerans*
NS-PDC-93	KT922562	*Lachancea thermotolerans*
NS-PDC-94	KT922563	*Lachancea thermotolerans*
NS-PDC-95	KT922564	*Lachancea thermotolerans*
NS-PDC-96	KT922565	*Lachancea thermotolerans*
NS-PDC-97	KT922566	*Lachancea thermotolerans*
NS-PDC-98	KT922567	*Lachancea thermotolerans*
NS-PDC-99	KT922568	*Kluyveromyces marxianus*
NS-PDC-100	KT922569	*Kluyveromyces marxianus*
NS-PDC-101	KT922570	*Hanseniaspora uvarum*
NS-PDC-102	KT922571	*Hanseniaspora uvarum*
NS-PDC-103	KT922572	*Hanseniaspora uvarum*
NS-PDC-104	KT922573	*Hanseniaspora uvarum*
NS-PDC-105	KT922574	*Hanseniaspora uvarum*
NS-PDC-106	KT922575	*Hanseniaspora uvarum*
NS-PDC-107	KT922576	*Hanseniaspora uvarum*
NS-PDC-108	KT922577	*Hanseniaspora uvarum*
NS-PDC-109	KT922578	*Hanseniaspora uvarum*
NS-PDC-110	KT922579	*Hanseniaspora uvarum*
NS-PDC-111	KT922580	*Hanseniaspora uvarum*
NS-PDC-112	KT922581	*Hanseniaspora uvarum*
NS-PDC-115	KT922582	*Hanseniaspora uvarum*
NS-PDC-116	KT922583	*Hanseniaspora uvarum*

NS-PDC-117	KT922584	*Hanseniaspora uvarum*
NS-PDC-118	KT922585	*Hanseniaspora uvarum*
NS-PDC-119	KT922586	*Hanseniaspora uvarum*
NS-PDC-120	KT922587	*Hanseniaspora uvarum*
NS-PDC-121	KT922588	*Aureobasidium pullulans*
NS-PDC-123	KT922589	*Aureobasidium pullulans*
NS-PDC-124	KT922590	*Aureobasidium pullulans*
NS-PDC-125	KT922591	*Aureobasidium pullulans*
NS-PDC-127	KT922592	*Aureobasidium pullulans*
NS-PDC-128	KT922593	*Aureobasidium pullulans*
NS-PDC-129	KT922594	*Aureobasidium pullulans*
NS-PDC-130	KT922595	*Aureobasidium pullulans*
NS-PDC-131	KT922596	*Aureobasidium pullulans*
NS-PDC-132	KT922597	*Cryptococcus amylolentus*
NS-PDC-133	KT922598	*Cryptococcus amylolentus*
NS-PDC-134	KT922599	*Aureobasidium pullulans*
NS-PDC-135	KT922600	*Aureobasidium pullulans*
NS-PDC-136	KT922601	*Aureobasidium pullulans*
NS-PDC-137	KT922602	*Aureobasidium pullulans*
NS-PDC-138	KT922603	*Aureobasidium pullulans*
NS-PDC-139	KT922604	*Aureobasidium pullulans*
NS-PDC-140	KT922605	*Aureobasidium pullulans*
NS-PDC-141	KT922606	*Metschnikowia sp.*
NS-PDC-142	KT922607	*Metschnikowia sp.*
NS-PDC-143	KT922608	*Metschnikowia sp.*
NS-PDC-144	KT922609	*Metschnikowia sp.*
NS-PDC-146	KT922610	*Metschnikowia sp.*
NS-PDC-147	KT922611	*Metschnikowia sp.*
NS-PDC-148	KT922612	*Metschnikowia sp.*
NS-PDC-149	KT922613	*Metschnikowia sp.*
NS-PDC-150	KT922614	*Metschnikowia sp.*
NS-PDC-151	KT922615	*Metschnikowia sp.*
NS-PDC-152	KT922616	*Metschnikowia sp.*
NS-PDC-153	KT922617	*Metschnikowia sp.*
NS-PDC-154	KT922618	*Metschnikowia sp.*
NS-PDC-155	KT922619	*Metschnikowia sp.*
NS-PDC-156	KT922620	*Metschnikowia sp.*
NS-PDC-157	KT922621	*Metschnikowia sp.*
NS-PDC-158	KT922622	*Metschnikowia sp.*
NS-PDC-159	KT922623	*Metschnikowia sp.*
NS-PDC-160	KT922624	*Metschnikowia sp.*
NS-PDC-161	KT922625	*Aureobasidium pullulans*
NS-PDC-162	KT922626	*Hanseniaspora uvarum*
NS-PDC-163	KT922627	*Hanseniaspora uvarum*
NS-PDC-164	KT922628	*Hanseniaspora uvarum*
NS-PDC-165	KT922629	*Aureobasidium pullulans*

PDC (2014)	NS-PDC-166	KT922630	*Hanseniaspora uvarum*
	NS-PDC-167	KT922631	*Wickerhamomyces anomalus*
	NS-PDC-168	KT922632	*Hanseniaspora uvarum*
	NS-PDC-169	KT922633	*Torulaspora delbrueckii*
	NS-PDC-170	KT922634	*Hanseniaspora uvarum*
	NS-PDC-171	KT922635	*Wickerhamomyces anomalus*
	NS-PDC-172	KT922636	*Aureobasidium pullulans*
	NS-PDC-173	KT922637	*Aureobasidium pullulans*
	NS-PDC-174	KT922638	*Lachancea thermotolerans*
	NS-PDC-175	KT922639	*Hanseniaspora uvarum*
	NS-PDC-176	KT922640	*Metschnikowia sp.*
	NS-PDC-177	KT922641	*Metschnikowia sp.*
	NS-PDC-178	KT922642	*Cryptococcus amylolentus*
	NS-PDC-179	KT922643	*Metschnikowia sp.*
	NS-PDC-180	KT922644	*Metschnikowia sp.*
	NS-PDC-181	KT922645	*Hanseniaspora uvarum*
	NS-PDC-182	KT922646	*Hanseniaspora uvarum*
	NS-PDC-183	KT922647	*Hanseniaspora uvarum*
	NS-PDC-184	KT922648	*Hanseniaspora uvarum*
	NS-PDC-185	KT922649	*Hanseniaspora uvarum*
	NS-PDC-186	KT922650	*Hanseniaspora uvarum*
	NS-PDC-187	KT922651	*Hanseniaspora uvarum*
	NS-PDC-188	KT922652	*Hanseniaspora uvarum*
	NS-PDC-189	KT922653	*Hanseniaspora uvarum*
	NS-PDC-190	KT922654	*Hanseniaspora uvarum*
	NS-PDC-191	KT922655	*Metschnikowia sp.*
	NS-PDC-192	KT922656	*Metschnikowia sp.*
	NS-PDC-193	KT922657	*Metschnikowia sp.*
	NS-PDC-194	KT922658	*Metschnikowia sp.*
	NS-PDC-195	KT922659	*Metschnikowia sp.*
	NS-PDC-196	KT922660	*Metschnikowia sp.*
	NS-PDC-197	KT922661	*Metschnikowia sp.*
	NS-PDC-198	KT922662	*Metschnikowia sp.*
	NS-PDC-199	KT922663	*Metschnikowia sp.*
	NS-PDC-200	KT922664	*Metschnikowia sp.*
	NS-PDC-201	KT922665	*Metschnikowia sp.*
	NS-PDC-202	KT922666	*Metschnikowia sp.*
	NS-PDC-205	KT922667	*Lachancea thermotolerans*
	NS-PDC-206	KT922668	*Metschnikowia sp.*
	NS-PDC-207	KT922669	*Metschnikowia sp.*
	NS-PDC-208	KT922670	*Metschnikowia sp.*
	NS-PDC-209	KT922671	*Hanseniaspora uvarum*
	NS-PDC-210	KT922672	*Hanseniaspora uvarum*
	NS-PDC-211	KT922673	*Hanseniaspora uvarum*
	NS-PDC-212	KT922674	*Hanseniaspora uvarum*
	NS-PDC-213	KT922675	*Metschnikowia sp.*

	NS-PDC-214	KT922676	*Metschnikowia sp.*
	NS-PDC-215	KT922677	*Metschnikowia sp.*
	NS-PDC-217	KT922678	*Metschnikowia sp.*
	NS-PDC-218	KT922679	*Hanseniaspora uvarum*
	NS-PDC-219	KT922680	*Metschnikowia sp.*
	NS-PDC-220	KT922681	*Metschnikowia sp.*
	NS-PDC-221	KT922682	*Hanseniaspora uvarum*
	NS-PDC-222	KT922683	*Hanseniaspora uvarum*
	NS-PDC-223	KT922684	*Hanseniaspora uvarum*
	NS-PDC-224	KT922685	*Hanseniaspora uvarum*
	NS-PDC-225	KT922686	*Hanseniaspora uvarum*
	NS-PDC-226	KT922687	*Hanseniaspora uvarum*
	NS-PDC-227	KT922688	*Hanseniaspora uvarum*
	NS-PDC-228	KT922689	*Hanseniaspora uvarum*
	NS-PDC-229	KT922690	*Hanseniaspora uvarum*
	NS-PDC-230	KT922691	*Hanseniaspora uvarum*
	NS-PDC-231	KT922692	*Hanseniaspora uvarum*
	NS-PDC-232	KT922693	*Hanseniaspora uvarum*
	NS-PDC-233	KT922694	*Hanseniaspora uvarum*
	NS-PDC-234	KT922695	*Hanseniaspora uvarum*
	NS-PDC-235	KT922696	*Hanseniaspora uvarum*
	NS-PDC-236	KT922697	*Hanseniaspora uvarum*
	NS-PDC-237	KT922698	*Hanseniaspora uvarum*
	NS-PDC-238	KT922699	*Hanseniaspora uvarum*
	NS-PDC-239	KT922700	*Hanseniaspora uvarum*
	NS-PDC-240	KT922701	*Hanseniaspora uvarum*
	NS-PDC-241	KT922702	*Metschnikowia sp.*
	NS-PDC-242	KT922703	*Cryptococcus amylolentus*
	NS-PDC-243	KT922704	*Cryptococcus amylolentus*
	NS-PDC-244	KT922705	*Cryptococcus amylolentus*
	NS-PDC-245	KT922706	*Cryptococcus amylolentus*
	NS-PDC-246	KT922707	*Cryptococcus amylolentus*
	NS-PDC-247	KT922708	*Metschnikowia sp.*
	NS-PDC-248	KT922709	*Cryptococcus amylolentus*
	NS-PDC-249	KT922710	*Cryptococcus amylolentus*
	NS-PDC-250	KT922711	*Cryptococcus amylolentus*
	NS-PDC-251	KT922712	*Metschnikowia sp.*
	NS-PDC-252	KT922713	*Cryptococcus amylolentus*
	NS-PDC-253	KT922714	*Cryptococcus amylolentus*
	NS-PDC-254	KT922715	*Cryptococcus amylolentus*
	NS-PDC-255	KT922716	*Metschnikowia sp.*
	NS-PDC-256	KT922717	*Metschnikowia sp.*
	NS-PDC-257	KT922718	*Cryptococcus amylolentus*
	NS-PDC-258	KT922719	*Metschnikowia sp.*
	NS-PDC-259	KT922720	*Metschnikowia sp.*
	NS-PDC-260	KT922721	*Metschnikowia sp.*

	NS-PDC-261	KT922722	*Cryptococcus amylolentus*
	NS-PDC-262	KT922723	*Cryptococcus amylolentus*
	NS-EM-1	KT922276	*Hanseniaspora uvarum*
	NS-EM-2	KT922277	*Hanseniaspora uvarum*
	NS-EM-3	KT922278	*Hanseniaspora uvarum*
	NS-EM-4	KT922279	*Hanseniaspora uvarum*
	NS-EM-5	KT922280	*Hanseniaspora uvarum*
	NS-EM-6	KT922281	*Hanseniaspora uvarum*
	NS-EM-7	KT922282	*Hanseniaspora uvarum*
	NS-EM-8	KT922283	*Hanseniaspora uvarum*
	NS-EM-9	KT922284	*Hanseniaspora uvarum*
	NS-EM-10	KT922285	*Hanseniaspora uvarum*
	NS-EM-12	KT922286	*Hanseniaspora uvarum*
	NS-EM-13	KT922287	*Hanseniaspora uvarum*
	NS-EM-14	KT922288	*Hanseniaspora uvarum*
	NS-EM-15	KT922289	*Metschnikowia sp.*
	NS-EM-16	KT922290	*Hanseniaspora uvarum*
	NS-EM-17	KT922291	*Hanseniaspora uvarum*
	NS-EM-18	KT922292	*Hanseniaspora uvarum*
	NS-EM-19	KT922293	*Hanseniaspora uvarum*
	NS-EM-20	KT922294	*Hanseniaspora uvarum*
	NS-EM-21	KT922295	*Hanseniaspora uvarum*
	NS-EM-22	KT922296	*Hanseniaspora uvarum*
	NS-EM-23	KT922297	*Hanseniaspora uvarum*
	NS-EM-24	KT922298	*Hanseniaspora uvarum*
	NS-EM-25	KT922299	*Hanseniaspora uvarum*
	NS-EM-26	KT922300	*Hanseniaspora uvarum*
	NS-EM-27	KT922301	*Hanseniaspora uvarum*
	NS-EM-28	KT922302	*Hanseniaspora uvarum*
	NS-EM-29	KT922303	*Hanseniaspora uvarum*
	NS-EM-30	KT922304	*Hanseniaspora uvarum*
	NS-EM-31	KT922305	*Hanseniaspora uvarum*
	NS-EM-32	KT922306	*Hanseniaspora uvarum*
	NS-EM-33	KT922307	*Hanseniaspora uvarum*
	NS-EM-34	KT222665	*Metschnikowia sp.*
	NS-EM-36	KT922308	*Hanseniaspora uvarum*
	NS-EM-37	KT922309	*Hanseniaspora uvarum*
	NS-EM-38	KT922310	*Hanseniaspora uvarum*
	NS-EM-39	KT922311	*Hanseniaspora uvarum*
	NS-EM-40	KT922312	*Hanseniaspora uvarum*
	NS-EM-41	KT922313	*Hanseniaspora uvarum*
	NS-EM-42	KT922314	*Hanseniaspora uvarum*
	NS-EM-43	KT922315	*Hanseniaspora uvarum*
	NS-EM-44	KT922316	*Hanseniaspora uvarum*
	NS-EM-45	KT922317	*Hanseniaspora uvarum*
	NS-EM-46	KT922318	*Hanseniaspora uvarum*

EM (2013)	NS-EM-47	KT922319	*Hanseniaspora uvarum*
	NS-EM-48	KT922320	*Hanseniaspora uvarum*
	NS-EM-49	KT922321	*Hanseniaspora uvarum*
	NS-EM-50	KT922322	*Hanseniaspora uvarum*
	NS-EM-51	KT922323	*Lachancea thermotolerans*
	NS-EM-52	KT922324	*Lachancea thermotolerans*
	NS-EM-53	KT922325	*Lachancea thermotolerans*
	NS-EM-54	KT922326	*Lachancea thermotolerans*
	NS-EM-55	KT922327	*Lachancea thermotolerans*
	NS-EM-56	KT922328	*Lachancea thermotolerans*
	NS-EM-57	KT922329	*Lachancea thermotolerans*
	NS-EM-58	KT922330	*Lachancea thermotolerans*
	NS-EM-59	KT922331	*Lachancea thermotolerans*
	NS-EM-60	KT922332	*Lachancea thermotolerans*
	NS-EM-61	KT922333	*Lachancea thermotolerans*
	NS-EM-62	KT922334	*Lachancea thermotolerans*
	NS-EM-63	KT922335	*Lachancea thermotolerans*
	NS-EM-64	KT922336	*Lachancea thermotolerans*
	NS-EM-65	KT922337	*Lachancea thermotolerans*
	NS-EM-66	KT922338	*Lachancea thermotolerans*
	NS-EM-67	KT922339	*Lachancea thermotolerans*
	NS-EM-68	KT922340	*Lachancea thermotolerans*
	NS-EM-69	KT922341	*Lachancea thermotolerans*
	NS-EM-70	KT922342	*Lachancea thermotolerans*
	NS-EM-71	KT922343	*Lachancea thermotolerans*
	NS-EM-72	KT922344	*Lachancea thermotolerans*
	NS-EM-73	KT922345	*Lachancea thermotolerans*
	NS-EM-74	KT922346	*Lachancea thermotolerans*
	NS-EM-75	KT922347	*Lachancea thermotolerans*
	NS-EM-76	KT922348	*Hanseniaspora uvarum*
	NS-EM-77	KT922349	*Hanseniaspora uvarum*
	NS-EM-78	KT922350	*Hanseniaspora uvarum*
	NS-EM-80	KT922351	*Hanseniaspora uvarum*
	NS-EM-81	KT922352	*Hanseniaspora uvarum*
	NS-EM-82	KT922353	*Hanseniaspora uvarum*
	NS-EM-83	KT922354	*Hanseniaspora uvarum*
	NS-EM-84	KT922355	*Hanseniaspora uvarum*
	NS-EM-85	KT922356	*Hanseniaspora uvarum*
	NS-EM-86	KT922357	*Hanseniaspora uvarum*
	NS-EM-87	KT922358	*Hanseniaspora uvarum*
	NS-EM-88	KT922359	*Hanseniaspora uvarum*
	NS-EM-89	KT922360	*Hanseniaspora uvarum*
	NS-EM-90	KT922361	*Hanseniaspora uvarum*
	NS-EM-91	KT922362	*Hanseniaspora uvarum*
	NS-EM-92	KT922363	*Hanseniaspora uvarum*
	NS-EM-93	KT922364	*Hanseniaspora uvarum*

NS-EM-94	KT922365	*Hanseniaspora uvarum*
NS-EM-95	KT922366	*Hanseniaspora uvarum*
NS-EM-96	KT922367	*Hanseniaspora uvarum*
NS-EM-97	KT922368	*Hanseniaspora uvarum*
NS-EM-98	KT922369	*Hanseniaspora uvarum*
NS-EM-99	KT922370	*Hanseniaspora uvarum*
NS-EM-100	KT922371	*Hanseniaspora uvarum*
NS-EM-101	KT922372	*Hanseniaspora uvarum*
NS-EM-102	KT922373	*Hanseniaspora uvarum*
NS-EM-103	KT922374	*Hanseniaspora uvarum*
NS-EM-104	KT922375	*Lachancea thermotolerans*
NS-EM-105	KT922376	*Hanseniaspora uvarum*
NS-EM-106	KT922377	*Hanseniaspora uvarum*
NS-EM-107	KT922378	*Hanseniaspora uvarum*
NS-EM-108	KT922379	*Hanseniaspora uvarum*
NS-EM-109	KT922380	*Hanseniaspora uvarum*
NS-EM-110	KT922381	*Hanseniaspora uvarum*
NS-EM-111	KT922382	*Metschnikowia sp.*
NS-EM-112	KT922383	*Hanseniaspora uvarum*
NS-EM-113	KT922384	*Metschnikowia sp.*
NS-EM-114	KT922385	*Hanseniaspora uvarum*
NS-EM-115	KT922386	*Metschnikowia sp.*
NS-EM-116	KT922387	*Hanseniaspora uvarum*
NS-EM-117	KT922388	*Hanseniaspora uvarum*
NS-EM-118	KT922389	*Hanseniaspora uvarum*
NS-EM-119	KT922390	*Lachancea thermotolerans*
NS-EM-120	KT922391	*Hanseniaspora uvarum*
NS-EM-121	KT922392	*Hanseniaspora uvarum*
NS-EM-122	KT922393	*Hanseniaspora uvarum*
NS-EM-123	KT922394	*Metschnikowia sp.*
NS-EM-124	KT922395	*Hanseniaspora uvarum*
NS-EM-125	KT922396	*Hanseniaspora uvarum*
NS-EM-126	KT922397	*Hanseniaspora uvarum*
NS-EM-127	KT922398	*Hanseniaspora uvarum*
NS-EM-128	KT922399	*Hanseniaspora uvarum*
NS-EM-129	KT922400	*Hanseniaspora uvarum*
NS-EM-130	KT922401	*Lachancea thermotolerans*
NS-EM-131	KT922402	*Hanseniaspora uvarum*
NS-EM-132	KT922403	*Hanseniaspora uvarum*
NS-EM-133	KT922404	*Hanseniaspora uvarum*
NS-EM-134	KT922405	*Hanseniaspora uvarum*
NS-EM-135	KT922406	*Hanseniaspora uvarum*
NS-EM-136	KT922407	*Lachancea thermotolerans*
NS-EM-137	KT922408	*Hanseniaspora uvarum*
NS-EM-138	KT922409	*Hanseniaspora uvarum*
NS-EM-139	KT922410	*Lachancea thermotolerans*

EM (2014)	NS-EM-140	KT922411	*Hanseniaspora uvarum*
	NS-EM-141	KT922412	*Lachancea thermotolerans*
	NS-EM-142	KT922413	*Hanseniaspora uvarum*
	NS-EM-143	KT922414	*Hanseniaspora uvarum*
	NS-EM-144	KT922415	*Hanseniaspora uvarum*
	NS-EM-145	KT922416	*Hanseniaspora uvarum*
	NS-EM-146	KT922417	*Hanseniaspora uvarum*
	NS-EM-147	KT922418	*Hanseniaspora uvarum*
	NS-EM-148	KT922419	*Hanseniaspora uvarum*
	NS-EM-149	KT922420	*Hanseniaspora uvarum*
	NS-EM-150	KT922421	*Hanseniaspora uvarum*
	NS-EM-151	KT922422	*Hanseniaspora uvarum*
	NS-EM-152	KT922423	*Hanseniaspora uvarum*
	NS-EM-153	KT922424	*Hanseniaspora uvarum*
	NS-EM-154	KT922425	*Hanseniaspora uvarum*
	NS-EM-155	KT922426	*Hanseniaspora uvarum*
	NS-EM-156	KT922427	*Hanseniaspora uvarum*
	NS-EM-157	KT922428	*Hanseniaspora uvarum*
	NS-EM-158	KT922429	*Hanseniaspora uvarum*
	NS-EM-159	KT922430	*Hanseniaspora uvarum*
	NS-EM-160	KT922431	*Hanseniaspora uvarum*
	NS-EM-161	KT922432	*Hanseniaspora uvarum*
	NS-EM-162	KT922433	*Hanseniaspora uvarum*
	NS-EM-163	KT922434	*Hanseniaspora uvarum*
	NS-EM-164	KT922435	*Hanseniaspora uvarum*
	NS-EM-165	KT922436	*Hanseniaspora uvarum*
	NS-EM-166	KT922437	*Hanseniaspora uvarum*
	NS-EM-167	KT922438	*Metschnikowia sp.*
	NS-EM-168	KT922439	*Hanseniaspora uvarum*
	NS-EM-169	KT922440	*Hanseniaspora uvarum*
	NS-EM-170	KT922441	*Hanseniaspora uvarum*
	NS-EM-171	KT922442	*Hanseniaspora uvarum*
	NS-EM-172	KT922443	*Metschnikowia sp.*
	NS-EM-173	KT922444	*Hanseniaspora uvarum*
	NS-EM-174	KT922445	*Hanseniaspora uvarum*
	NS-EM-175	KT922446	*Hanseniaspora uvarum*
	NS-EM-176	KT922447	*Hanseniaspora uvarum*
	NS-EM-177	KT922448	*Hanseniaspora uvarum*
	NS-EM-178	KT922449	*Hanseniaspora uvarum*
	NS-EM-179	KT922450	*Hanseniaspora uvarum*
	NS-EM-180	KT922451	*Hanseniaspora uvarum*
	NS-EM-181	KT922452	*Hanseniaspora uvarum*
	NS-EM-182	KT922453	*Hanseniaspora uvarum*
	NS-EM-183	KT922454	*Hanseniaspora uvarum*
	NS-EM-184	KT922455	*Metschnikowia sp.*
	NS-EM-186	KT922456	*Hanseniaspora uvarum*

NS-EM-187	KT922457	*Metschnikowia sp.*
NS-EM-188	KT922458	*Hanseniaspora uvarum*
NS-EM-189	KT922459	*Hanseniaspora uvarum*
NS-EM-190	KT922460	*Hanseniaspora uvarum*
NS-EM-191	KT922461	*Hanseniaspora uvarum*
NS-EM-192	KT922462	*Hanseniaspora uvarum*
NS-EM-193	KT922463	*Hanseniaspora uvarum*
NS-EM-194	KT922464	*Metschnikowia sp.*
NS-EM-195	KT922465	*Hanseniaspora uvarum*
NS-EM-196	KT922466	*Hanseniaspora uvarum*
NS-EM-197	KT922467	*Metschnikowia sp.*
NS-EM-198	KT922468	*Hanseniaspora uvarum*
NS-EM-199	KT922469	*Hanseniaspora uvarum*
NS-EM-200	KT922470	*Hanseniaspora uvarum*
NS-G-1	KT922970	*Hanseniaspora uvarum*
NS-G-2	KT922971	*Lachancea thermotolerans*
NS-G-3	KT922972	*Lachancea thermotolerans*
NS-G-4	KT922973	*Hanseniaspora opuntiae*
NS-G-5	KT922974	*Hanseniaspora opuntiae*
NS-G-6	KT922975	*Lachancea thermotolerans*
NS-G-7	KT922976	*Hanseniaspora opuntiae*
NS-G-8	KT922977	*Hanseniaspora opuntiae*
NS-G-9	KT922978	*Torulaspora delbrueckii*
NS-G-10	KT922979	*Hanseniaspora uvarum*
NS-G-11	KT922980	*Hanseniaspora uvarum*
NS-G-12	KT922981	*Hanseniaspora uvarum*
NS-G-13	KT922982	*Lachancea thermotolerans*
NS-G-14	KT922983	*Hanseniaspora uvarum*
NS-G-15	KT922984	*Hanseniaspora opuntiae*
NS-G-16	KT922985	*Hanseniaspora opuntiae*
NS-G-17	KT922986	*Hanseniaspora uvarum*
NS-G-18	KT922987	*Hanseniaspora uvarum*
NS-G-19	KT922988	*Hanseniaspora uvarum*
NS-G-20	KT922989	*Hanseniaspora opuntiae*
NS-G-21	KT922990	*Hanseniaspora uvarum*
NS-G-22	KT922991	*Hanseniaspora uvarum*
NS-G-23	KT922992	*Hanseniaspora opuntiae*
NS-G-24	KT922993	*Saccharomyces cerevisiae*
NS-G-25	KT922994	*Lachancea thermotolerans*
NS-G-26	KT922995	*Hanseniaspora uvarum*
NS-G-27	KT922996	*Torulaspora delbrueckii*
NS-G-28	KT922997	*Hanseniaspora uvarum*
NS-G-29	KT922998	*Hanseniaspora uvarum*
NS-G-30	KT922999	*Saccharomyces cerevisiae*
NS-G-31	KT923000	*Saccharomyces cerevisiae*
NS-G-32	KT222664	*Lachancea thermotolerans*

G (2012)	NS-G-33	KT923001	*Hanseniaspora osmophila*
	NS-G-34	KT923002	*Wickerhamomyces anomalus*
	NS-G-35	KT923003	*Hanseniaspora osmophila*
	NS-G-36	KT923004	*Hanseniaspora osmophila*
	NS-G-37	KT923005	*Saccharomyces cerevisiae*
	NS-G-38	KT923006	*Hanseniaspora osmophila*
	NS-G-39	KT923007	*Hanseniaspora osmophila*
	NS-G-40	KT923008	*Hanseniaspora osmophila*
	NS-G-41	KT923009	*Hanseniaspora osmophila*
	NS-G-42	KT923010	*Saccharomyces cerevisiae*
	NS-G-43	KT923011	*Hanseniaspora osmophila*
	NS-G-44	KT923012	*Saccharomyces cerevisiae*
	NS-G-45	KT923013	*Hanseniaspora osmophila*
	NS-G-46	KT923014	*Torulaspora delbrueckii*
	NS-G-47	KT923015	*Hanseniaspora osmophila*
	NS-G-48	KT923016	*Saccharomyces cerevisiae*
	NS-G-49	KT923017	*Hanseniaspora osmophila*
	NS-G-50	KT923018	*Saccharomyces cerevisiae*
	NS-G-51	KT923019	*Hanseniaspora osmophila*
	NS-G-52	KT923020	*Saccharomyces cerevisiae*
	NS-G-53	KT923021	*Hanseniaspora osmophila*
	NS-G-54	KT923022	*Saccharomyces cerevisiae*
	NS-G-55	KT923023	*Saccharomyces cerevisiae*
	NS-G-56	KT923024	*Hanseniaspora osmophila*
	NS-G-57	KT923025	*Meyerozyma guilliermondii*
	NS-G-58	KT923026	*Zygosaccharomyces baillii*
	NS-G-59	KT923027	*Hanseniaspora uvarum*
	NS-G-60	KT923028	*Hanseniaspora uvarum*
	NS-G-61	KT923029	*Rhodosporidium toruloides*
	NS-G-62	KT923030	*Torulaspora delbrueckii*
	NS-G-63	KT923031	*Zygosaccharomyces baillii*
	NS-G-64	KT923032	*Hanseniaspora uvarum*
	NS-G-65	KT923033	*Hanseniaspora uvarum*
	NS-G-66	KT923034	*Torulaspora delbrueckii*
	NS-G-67	KT923035	*Hanseniaspora uvarum*
	NS-G-68	KT923036	*Hanseniaspora uvarum*
	NS-G-69	KT923037	*Hanseniaspora uvarum*
	NS-G-70	KT923038	*Rhodosporidium toruloides*
	NS-G-71	KT923039	*Torulaspora delbrueckii*
	NS-G-72	KT923040	*Torulaspora delbrueckii*
	NS-G-73	KT923041	*Hanseniaspora uvarum*
