# Supplementary material for: Unraveling the Enzymatic Basis of Wine “Flavorome”: A Phylo-Functional Study of Wine Related Yeast Species
Source: Front Microbiol. 2016 Jan 20;7:12. doi: 10.3389/fmicb.2016.00012 (PMC4718978; doi:10.3389/fmicb.2016.00012)
Supplement: Supplementary file 2 [file Table2.PDF]

Supplementary material. Belda et al.

Unraveling the enzymatic basis of wine "flavorome": a phylo-functional study of wine related yeast species

Table S2a. Component scores of the PCA analysis of *Torulaspora delbrueckii* isolates.

	PC1	PC2	PC3	PC4
**PCA**	65,39%	19,84%	14,77%	0,00%
β-glucosidase	-2,7525972	-0,4350818	0,4836448	0
β-D-xylosidase	-0,454887	-0,2758188	-0,7694166	0
α-L-arabinofuranosidase	0,454887	0,2758188	0,7694166	0
β-lyase	0	0	0	0
Protease	0	0	0	0
Pectinase	-1,0191866	1,5443728	-0,2759914	0
Cellulase	0	0	0	0
Hydrogen sulfide	0,454887	0,2758188	0,7694166	0

Table S2b. Component scores of the PCA analysis of *Aureobasidium pullulans* isolates.

	PC1	PC2	PC3	PC4	PC5
**PCA**	81,84%	12,71%	3,96%	1,49%	0,00%
β-glucosidase	-3,9674513	-0,0657378	0,3954627	-0,3140354	0
β-D-xylosidase	2,9145225	0,0674117	0,6352186	-0,3708255	0
α-L-arabinofuranosidase	0,3608266	-0,0006769	-0,7884007	-0,45632	0
β-lyase	0	0	0	0	0
Protease	0	0	0	0	0
Pectinase	0	0	0	0	0
Cellulase	0	0	0	0	0
Hydrogen sulfide	0,2349292	-1,9454368	0,0089224	-0,0020793	0

Table S2c. Component scores of the PCA analysis of *Wickerhamomyces anomalus* isolates.

	PC1	PC2	PC3	PC4	PC5
**PCA**	63,88%	32,87%	1,67%	1,57%	0,00%
β-glucosidase	0,3119884	0,8279487	0,1125434	0,0670679	0
β-D-xylosidase	0,1701489	-1,3376974	0,4051581	0,1321433	0
α-L-arabinofuranosidase	-0,6239768	-1,6558975	-0,2250869	-0,1341357	0
β-lyase	-0,9734537	-0,1301428	-0,1692056	0,4547748	0
Protease	3,0021804	-0,3965892	-0,136305	0,1051224	0
Pectinase	0	0	0	0	0
Cellulase	0	0	0	0	0
Hydrogen sulfide	0	0	0	0	0

Table S2d. Component scores of the PCA analysis of *Lachancea thermotolerans* isolates.

	PC1	PC2	PC3	PC4	PC5	PC6
**PCA**	47,17%	32,11%	12,59%	7,54%	0,58%	0,00%
β-glucosidase	-2,7019002	-2,2142606	0,1526593	3,1838648	0,0110519	0
β-D-xylosidase	-6,0620138	-3,9149433	-1,269482	-1,4245284	-0,0158298	0
α-L-arabinofuranosidase	0,1343755	-0,0228551	0,0113386	0,0581786	-0,9831297	0
β-lyase	-3,4338728	0,8871775	4,1287642	-0,4391873	-0,0059975	0
Protease	0	0	0	0	0	0
Pectinase	0	0	0	0	0	0
Cellulase	0	0	0	0	0	0
Hydrogen sulfide	4,7074035	-5,6646144	1,464285	-0,3290471	0,0096475	0

Table S2e. Component scores of the PCA analysis of *Hanseniaspora uvarum* isolates.

	PC1	PC2	PC3	PC4	PC5	PC6	PC7	PC8
**PCA**	62,62%	16,86%	8,30%	6,90%	2,58%	2,43%	0,32%	0,00%
β-glucosidase	1,6052499	-1,2517011	-0,2888223	1,890322	-5,3872489	0,9172722	-0,0133567	0
β-D-xylosidase	10,202646	12,8537732	1,0154342	2,1261501	-0,0610447	0,044465	-0,0316283	0
α-L-arabinofuranosidase	-0,0271649	0,6111734	-0,4786401	-1,0740953	-1,1139738	-5,2980603	0,0130678	0
β-lyase	1,6205529	-2,3458289	-5,5246202	7,2533963	1,0370313	-0,6165931	-0,0059899	0
Protease	-4,0082486	-2,3418536	8,2548803	4,7039366	0,3929968	-0,6703662	0,0010808	0
Pectinase	-0,0563053	0,2368151	-0,0156737	0,0796928	-0,0304144	0,0417455	1,9804444	0
Cellulase	0	0	0	0	0	0	0	0
Hydrogen sulfide	25,3287538	-5,317611	1,2685261	-0,6968938	0,3605937	-0,1482685	0,0185575	0
