# Supplementary material for: Unraveling the Enzymatic Basis of Wine “Flavorome”: A Phylo-Functional Study of Wine Related Yeast Species
Source: Front Microbiol. 2016 Jan 20;7:12. doi: 10.3389/fmicb.2016.00012 (PMC4718978; doi:10.3389/fmicb.2016.00012)
Supplement: Supplementary file 3 [file Table3.PDF]

Supplementary material. Belda et al.

Unraveling the enzymatic basis of wine "flavorome": a phylo-functional study of wine related yeast species

Table S3. Dissimilarity matrix for the similarity found during vintages and wine appellations.

	EM 2013	EM 2014	PDC 2013	PDC 2014	O 2013	O 2014	G 2012
EM 2013	0.00						
EM 2014	0.20	0.00					
PDC 2013	0.24	0.36	0.00				
PDC 2014	0.66	0.58	0.58	0.00			
O 2013	0.53	0.45	0.45	0.34	0.00		
O 2014	0.27	0.11	0.45	0.51	0.37	0.00	
G 2012	0.63	0.65	0.63	0.68	0.69	0.71	0.00

Values in the matrix range from 0 to 1, with 0 representing no differentiation between samples and 1 meaning no similarity.
