# Supplementary material for: Unraveling the Enzymatic Basis of Wine “Flavorome”: A Phylo-Functional Study of Wine Related Yeast Species
Source: Front Microbiol. 2016 Jan 20;7:12. doi: 10.3389/fmicb.2016.00012 (PMC4718978; doi:10.3389/fmicb.2016.00012)
Supplement: Supplementary file 5 [file Image1.PDF]

Supplementary material. Belda et al.

Unraveling the enzymatic basis of wine "flavorome": a phylo-functional study of wine related yeast species

Figure S2. Wine appellations sampled in the study indicating geographical and climatic data.

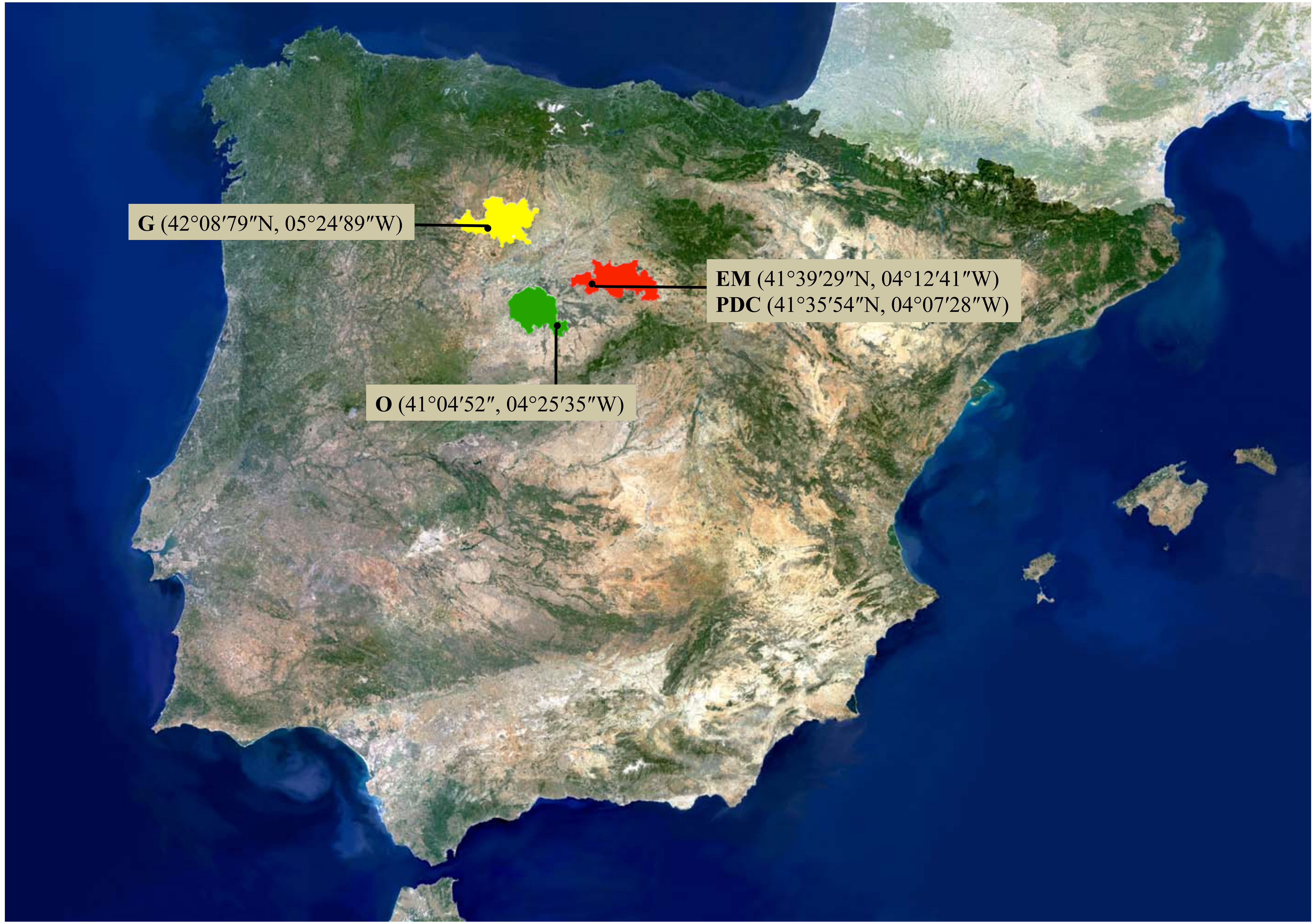

G vineyard
Wine apellation: Tierra de León
Grape variety: Prieto Picudo
Altitude: 747 m
Rainfall (total annual data): 224,9 mm (2012)
Mean maximum temperature: 18,5 ºC (2012)
Mean minimum temperature: 5,2 ºC (2012)

EM & PDC vineyards
Wine apellation: Ribera del Duero
Grape variety: Tempranillo
Altitude: 746 m (EM) & 754 m (PDC)
Rainfall (total annual data): 258,2 mm (2012) 444,8 mm (2013); 378,2 mm (2014)
Mean maximum temperature: 18,7 ºC (2012); 18 ºC (2013); 19,2 ºC (2014)
Mean minimum temperature: 5,4 ºC (2012); 5,7 ºC (2013); 7,1 ºC (2014)

O vineyards
Wine apellation: Rueda
Grape variety: Verdejo
Altitude: 842 m
Rainfall (total annual data): 280 mm (2012) 416,98 mm (2013); 350,7 mm (2014)
Mean maximum temperature: 19 ºC (2012); 18,6 ºC (2013); 19,1 ºC (2014)
Mean minimum temperature: 5,6 ºC (2012); 5,6 ºC (2013); 6,6 ºC (2014)
