# Supplementary material for: Unraveling the Enzymatic Basis of Wine “Flavorome”: A Phylo-Functional Study of Wine Related Yeast Species
Source: Front Microbiol. 2016 Jan 20;7:12. doi: 10.3389/fmicb.2016.00012 (PMC4718978; doi:10.3389/fmicb.2016.00012)

Supplementary material. Belda et al.
Unraveling the enzymatic basis of wine "flavorome": a phylo-functional study of wine related yeast species.

Figure S1. Phylogenetic tree for the sequences obtained of the 26S rRNA gene for the 770 isolates described in this work. In addition, the activity of these isolates for seven enzymatic activities with enological relevance was also included. The 8 enzymatic activities evaluated were: A) β-glucosidase; B) β-D-xylosidase; C) α-L-arabinofuranosidase; D) β-lyase; E) Protease; F) Polygalacturonase; G) Cellulase; H) Hydrogen sulfide production.

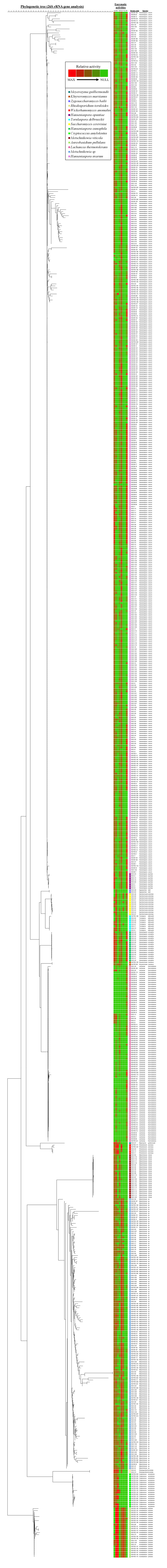

Supplement: Supplementary file 4 [file DataSheet1.PDF]
